# ER-to-Golgi Transport in HeLa Cells Displays High Resilience to Ca^2+^ and Energy Stresses

**DOI:** 10.3390/cells9102311

**Published:** 2020-10-17

**Authors:** Thomas Rauter, Sandra Burgstaller, Benjamin Gottschalk, Jeta Ramadani-Muja, Helmut Bischof, Jesse C. Hay, Wolfgang F. Graier, Roland Malli

**Affiliations:** 1 Molecular Biology and Biochemistry, Gottfried Schatz Research Center, Medical University of Graz, Neue Stiftingtalstraße 6/6, 8010 Graz, Austria; thomas.rauter@medunigraz.at (T.R.); sandra.burgstaller@uni-tuebingen.de (S.B.); benjamin.gottschalk@medunigraz.at (B.G.); jeta.ramadani@medunigraz.at (J.R.-M.); helmut.bischof@uni-tuebingen.de (H.B.); wolfgang.graier@medunigraz.at (W.F.G.); 2 Interfaculty Institute of Cell Biology, University of Tuebingen, Auf der Morgenstelle 15, 72076 Tuebingen, Germany; 3 Department of Pharmacology, Toxicology and Clinical Pharmacy, Institute of Pharmacy, University of Tuebingen, Auf der Morgenstelle 8, 72076 Tuebingen, Germany; 4 Division of Biological Sciences and Center for Structural and Functional Neuroscience, The University of Montana, 32 Campus Drive, HS 302A, Missoula, MT 59812-4824, USA; Jesse.Hay@mso.umt.edu; 5 BioTechMed Graz, Mozartgasse 12/II, 8010 Graz, Austria

**Keywords:** ER-to-Golgi transport, coat protein complex II (COPII) vesicles, vesicle trafficking, cancer cell metabolism, cellular calcium homeostasis, fluorescent protein technology, live-cell imaging, protein transport and sorting, secretory pathway, subcellular ATP imaging

## Abstract

One third of all human proteins are either transmembrane or soluble secretory proteins that first target the endoplasmic reticulum (ER). These proteins subsequently leave the ER and enter the Golgi apparatus via ER-Golgi intermediate vesicular structures. Live-cell imaging of cargos fused to fluorescent proteins (FPs) enables the high-resolution visualization and characterization of secretory transport processes. Here, we performed fluorescence time-lapse imaging to assess the Ca^2+^ and energy dependency of ER-to-Golgi transport in living HeLa cells, a cancer cell model which has been well investigated. Our data revealed that ER-to-Golgi transport remained highly efficient in the absence of ATP-generating substrates, despite clear reductions in cytosolic and mitochondrial ATP levels under these energy stress conditions. However, cell treatment with 2-deoxy-D-glucose (2-DG), which severely diminished subcellular ATP levels, abolished ER-to-Golgi transport. Interestingly, while 2-DG elevated cytosolic Ca^2+^ levels and reduced long-distance movements of glycosylphosphatidylinositol (GPI)-positive vesicles, robust short-term ER Ca^2+^ mobilizations, which strongly affected the motility of these vesicles, did not considerably impair ER-to-Golgi transport. In summary, we highlight that ER-to-Golgi transport in HeLa cells remains functional despite high energy and Ca^2+^ stress levels.

## 1. Introduction

The secretion of proteins from eukaryotic cells is a tightly regulated vital process based on complex membrane trafficking pathways [1]. Depending upon cell needs, protein secretion can reach extremely high levels and requires huge expansions of the endoplasmic reticulum (ER) [2], the entry gate to the secretory pathway, and of the Golgi apparatus, the key sorting and posttranslational modification hub in the secretory pathway. Up to one third of all proteins in eukaryotic cells enter the secretory pathway [3,4,5,6,7]. Strikingly, individual cells can secrete thousands of protein molecules per second [8]. Given these high rates of secretory activity and the overall importance of this process, it is not surprising that defects of the secretory pathway and membrane trafficking can impair cell functions and cause severe disease [1,7,9,10,11,12,13]. Protein secretion in cancer cells is crucial for malignancy [3,14,15,16,17,18,19]. A number of proteins trafficked through the secretory pathway of cancer cells are required to maintain uncontrolled proliferation, protect cancer cells from immune cells, and induce angiogenesis [3,15,17,19]. Thus, the secretory pathway represents a promising target in cancer therapy [3,15].

Research over the last 50 years has revealed that the secretory pathway was based on vesicular tubular clusters of transport carriers that moved from the rough ER to the Golgi apparatus, and then again from Golgi to the plasma membrane [1,20,21,22,23,24,25,26,27,28]. The key components of the ER-to-Golgi transport machinery are well identified [1]. Their regulators, function, and characteristics have been well investigated in different cell types and species and a number of required mechanisms have been studied extensively [29]. In addition to the anterograde pathway, mechanisms of retrograde Golgi-ER transport have also been described [30]. Interestingly, the main steps in ER-to-Golgi transport are conserved among different species [31], while there are some variations in transport carriers and the involvement of the cytoskeleton [26]. In mammalian cells, vesicular coat protein complex COPII-coated carriers bud at special ER-exit sites (ERES) on the ER surface [20,21,22]. COPII vesicles released from the ER are thought to form larger vesicular tubular clusters (VTCs) by homotypic fusion events [32,33]. These clusters partially build the ER-Golgi intermediate compartment (ERGIC), an intermediate organelle with static, as well as dynamic characteristics, before the cargo is shuttled to the cis-Golgi within vesicular structures [23,24,25,28]. The transport steps from the ER to the Golgi apparatus have been shown to work in a microtubule-dependent manner [24,25,26,28]. It is assumed that several steps of the secretory pathway are energy dependent [7]. Some older reports have demonstrated that the inhibition of ATP generation could strongly impact the secretory transport of particular cargo proteins [34,35,36,37]. While a mutual interrelation between the secretory rate and metabolic activity is frequently discussed [38,39], studies that aim at characterizing such crosstalk and its dynamics are rare. However, recent advances in the development of high-resolution live-cell imaging tools and techniques [38] have opened the door for meaningful experiments that could improve our understanding of metabolic implications in signaling and protein trafficking. Such modern approaches also allow one to conduct single cancer cell profiling [40], which might help to further develop personalized diagnostics and therapy in the battle against cancer.

At least in part, due to genetically encoded tools enabling transport visualization and subcellular Ca^2+^ measurements, recent data have confirmed the importance of global and local Ca^2+^ signals in regulating membrane trafficking for ER-to-Golgi transport, as well as exocytosis [41,42,43,44]. Since Ca^2+^ storage and extrusion is dependent on the amount of available ATP [45,46], any changes in metabolic activity can consequently impact the secretory pathway. Genetically encoded ATP sensors allow the real-time quantification of subcellular ATP levels [40,47,48] in correlation with respective changes of Ca^2+^ signals to estimate the actual impact of energy stress on the cellular Ca^2+^ homeostasis in individual cells. The green fluorescent protein (GFP) technology has also been exploited to visualize ER-to-Golgi transport at the single-cell level [25,33,49,50,51]. To synchronize the release of GFP-labeled cargo of interest from the ER, ligand-sensitive conditional aggregation domains (CADs) can be fused to the cargo [51,52,53]. In the absence of the ligand, CAD aggregations prevent the exit of the fluorescent construct from the ER. Upon ligand addition, the subsequent disaggregation immediately triggers the synchronized release of the GFP-tagged cargo construct into the secretory pathway.

In this study, we exploited these and other genetically encoded fluorescent biosensors to investigate the impact of energy and Ca^2+^ stress on the dynamics of ER-to-Golgi transport at the single-cell level using HeLa cells as a cell model for a highly malignant solid tumor with a cancer-specific metabolic setting [40], well-known as the Warburg effect [54,55,56]. Given the particular importance of the secretory pathway for cancer cell growth and viability, this study further aims to reveal whether ER-to-Golgi transport represents a sensitive target susceptible to Ca^2+^ and energy stresses. Strikingly, our data emphasize that ER-to-Golgi transport is high in HeLa cells and displays high resilience to short-term stresses.

## 2. Materials and Methods

### 2.1. Chemicals, Buffers, and Solutions

All salt components needed for the preparation of the measurement buffer, NaCl, KCl, MgCl_2_, CaCl_2_ as well as 4-(2-hydroxyethyl)-1-piperazineethanesulfonic acid (HEPES), D(+)-glucose monohydrate, adenosine-5′-triphosphate disodium salt (ATPNa_2_), 2,5-di-t-butyl-1,4-benzohydroquinone (BHQ), dimethylsulfoxide (DMSO), yeast extract, trypton/pepton from casein, agar-agar (Kobe I), and ethylene glycol-bis(β-aminoethyl ether)-N,N,N′,N′-tetraacetic acid (EGTA) were purchased from Carl Roth (Graz, Austria). The 2-deoxy-D-glucose (2-DG) was obtained from Alfa Aesar (Kandel, Germany). Agarose was acquired from VWR International (Vienna, Austria) and D/D Solubilizer was purchased from Takara (cat. no. 635054, St Germain-en-Laye, France). Prior to measurements, directly after getting the cells out of the incubator (37 °C, 5% CO_2_), cells were washed and stored for 45 min in storage buffer, which was composed of 138 mM NaCl, 5 mM KCl, 2 mM CaCl_2_, 1 mM MgCl_2_, 10 mM HEPES, 10 mM glucose, 2.5 mM L-glutamine, 2.6 mM NaHCO_3_, 0.34 mM Na_2_HPO_4_ (all, Carl Roth, Graz, Austria), 0.44 mM KH_2_PO_4_ (Merck, Darmstadt, Germany), 0.1% vitamins (Gibco MEM vitamin solution, Thermo Fisher Scientific, Vienna, Austria), 0.2% essential amino acids (Gibco MEM amino acid solution, Thermo Fisher Scientific, Vienna Austria), 1% penicillin-streptomycin and 0.5% amphothericin B (both, Thermo Fisher Scientific, Vienna, Austria); pH adjusted to 7.4 with NaOH. The measurement buffer with physiological pH of 7.4 (adjusted with NaOH) and isotonic salt composition was composed of 2 mM CaCl_2_, 5 mM KCl, 138 mM NaCl, 1 mM MgCl_2_, and 10 mM HEPES. The glucose-containing control buffer contained 10 mM glucose. The 2-DG buffer contained 10 mM 2-DG and no glucose. The calcium-free buffer was made the same way, except with no CaCl_2_ and, instead, 0.1 mM EGTA was added.

### 2.2. Cell Culture and Transfection

HeLa and NRK cells were obtained from ATCC (Guernsey, UK). As a standard medium for HeLa and NRK cells, DMEM (purchased from Sigma-Aldrich, Vienna, Austria), 10% fetal bovine serum, 100 U/mL penicillin, 100 μg/mL streptomycin, and 2.5 μg/mL Fungizone (all, Thermo Fisher Scientific, Vienna, Austria) were used. For transfection of these cells, the reagents Polyjet In Vitro Transfection Reagent (SignaGen, Rockville, MD, USA) or TransFast Transfection Reagent (Promega, Mannheim, Germany) for plasmid DNA were applied, according to the manufacturer’s protocols. In brief, 150,000 cells were seeded on 30 mm 1.5 H high-precision glass cover slides (MarienfeldSuperior, for SIM) positioned in 6-well plates and transfected on the following day at a confluency of 60–70%. The PolyJet transfection reagent was used 24 h prior to measurements. Cells were transfected with 1 µg DNA and incubated with the PolyJet-plasmid DNA mixture for 24–36 h. All cells were maintained in a humidified incubator (37 °C, 5% CO_2_, 95% air). The experiments were performed 24–36 h after transfection. 

### 2.3. Measuring Subcellular ATP and Ca^2+^ Dynamics Using Genetically Encoded Fluorescent Biosensors

ATP and Ca^2+^ measurements were conducted on an inverted fluorescence microscope, which was based on an IX73 Olympus stage (IX73 system, Vienna, Austria). As the excitation LED-based light source, the Omicron LEDHub (Omicron-Laserage, Dudenhof, Germany) was used and controlled by the VisiView software (Visiview v4.2.0.1, Visitron Systems GmbH, Puchheim, Germany, https://www.visitron.de/products/visiviewr-software.html). Imaging was performed with a 40× magnification oil immersion objective (UPLXAPO40XO, NA 1.4; Olympus, Vienna, Austria) and an optical beam splitter (Photometrics DV2, Photometrics, Tucson, AZ, USA) connected to a Retiga R1 CCD camera (TELEDYNE QIMAGING, Surrey, Canada). The filter system for CFP and Förster resonance energy transfer (FRET) measurements consisted of a Semrock 427/10 Brightline HC excitation filter and a Semrock LED-CFP/YFP/mCherry-3x emission filter (both, IDEX Health & Science LLC, Rochester, NY, USA). FURA-2 was alternately excited at 340 and 385 nm, and emission was collected at 515 nm. 

Prior to measurements, directly after getting the cells out of the incubator, cells were washed and stored for 45 min in storage buffer (see Section 2.1). After that, the cells grown on 30 mm glass coverslips were mounted into a PC30 perfusion chamber (NGFI GmbH, Graz, Austria). A stable baseline was recorded for 5 min while perfusing with measurement buffer with 10 mM glucose (see Section 2.1), using a gravity-based perfusion system for optimal buffer exchange (NGFI GmbH, Graz, Austria). Buffer removal was conducted with a Chemistry diaphragm vacuum pump ME 1c (Vacuubrand, Wertheim, Germany). For measuring ATP, we switched to buffer containing no glucose or 10 mM 2-DG, respectively, and kept perfusing with starting buffer for 60 min after that. Finally, all samples were perfused with buffer containing 10 mM 2-DG. For calcium measurements, we made use of the IP3-generating agonist ATP and BHQ, a reversible inhibitor of (sarco)endoplasmatic Ca^2+^ ATPase (SERCA). To our knowledge, BHQ acts specifically as a SERCA inhibitor in HeLa cells. After recording basal values, cells were perfused for 30 min with calcium-free buffer (see Section 2.1) containing 100 µM ATP and 15 µM BHQ, before switching back to starting buffer for another 15 min. Experiments aiming at retaining high cytosolic Ca^2+^ levels required perfusion with ATP/BHQ-containing starting buffer (2 mM Ca^2+^).

### 2.4. Live Cell Imaging of Endoplasmic Reticulum (ER)-to-Golgi Transport

Transport experiments and vesicle movement approaches were performed at a Zeiss array confocal laser scanning microscope (ACLSM, Zeiss Axiovert 200 M) using a 100×/1.45 oil immersion objective (Zeiss Microsystems, Jena, Germany). Illumination was conducted at 445 nm (pH-Lemon-glycosylphosphatidylinositol (GPI)) or 488 nm (GFP transport constructs) with an argon ion laser system (series 543, CVI Melles Griot, CA, USA) and emissions were collected at a binning of 2 with a CCD camera (CoolSnap HQ2; Photometrics, Tucson, Arizona, USA). Image analysis was performed in MetaMorph (Molecular devices) and ImageJ open source imaging analysis software (https://fiji.sc/) that was used to calculate quantification values for transport and vesicle movement experiments. The data analyses were conducted in Excel (Microsoft) and GraphPad Prism Software (Versions 5 and 8, GraphPad Software, Inc., La Jolla, CA, USA).

To measure secretory transport, we used two GFP-tagged constructs, GFP-F_M_4-GH (soluble cargo construct) and GFP-F_M_4-VSVG_tm_ (transmembrane cargo construct), which were kindly provided by Andrew Peden’s lab. These constructs formed aggregates in the ER lumen (GFP-F_M_4-GH) or at the ER membrane (GFP-F_M_4-VSVG_tm_), which prevented them from entering the secretory pathway. Upon addition of a ligand (“solubilizer”, 250 nM D/D-Solubilizer, cat. no. 635045, Takarabio, 78100 Saint-Germain-en-Laye, France), the protein aggregates disaggregated and transport was initiated. Constructs are described in more detail in Sargeant et al. 2020 [41]. The cells were co-transfected with one of the mentioned transport constructs and mCherry-Golgi-7 (excited at 561 nm), which helped define the Golgi apparatus location in the transport quantification. The mCherry-Golgi-7 was a gift from Michael Davidson (Addgene plasmid #55052, http://n2t.net/addgene:55052, RRID: Addgene_55052). After a 3 × 10 min pretreatment phase (either + glucose or − glucose, + 2-DG, respectively) or pretreatment of 15 min zero Ca^2+^ plus EGTA (0.1 mM) or 2 mM Ca^2+^ with added ATP (100 µM) and BHQ (15 µM) with one washing step, imaging was started. At time point 0, transport was initiated with D/D-Solubilizer injected to a final concentration of 250 nM and images were taken 3, 7, 15, and 30 min after that based on empirical testing.

The quantification procedure was carried out with a custom-made ImageJ macro, enabling semi-automated image analysis. Regions of interest (ROIs) were captured manually. The Otsu thresholding method, followed by two eroding steps, was applied on the RFP-images representing the Golgi region after subtracting background with a rolling ball and a binary image was created out of the resulting images. This procedure created a respective Golgi mask for each GFP image showing the transport construct, and it allowed a separation into Golgi and non-Golgi region. The integrated densities of these two regions, in the GFP channel, were measured automatically and a ratio (intensityGolgi/intensityNONGolgi) was calculated. For each cell the ratios of each time point were normalized to the first time point (transport initiation by adding D/D-Solubilizer). We named the result the “transport index” (“TI” = normalized intensityGolgi/intensityNONGolgi), i.e., a variable starting at 1 and increasing over time in case the Golgi/non-Golgi intensity ratio increases. As this ratio change is associated with decreased signal in the ER region and/or increased amount of GFP in the Golgi region, it can be interpreted as ER-to-Golgi transport occurring over time. 

### 2.5. Live Cell Imaging of Vesicle Movements

HeLa cells stably expressing pH-Lemon-GPI [57,58] were used to investigate vesicle movement. Pretreatment (+ glucose, − glucose, + 2-DG, EGTA/2Ca + ATP + BHQ) was conducted equivalent to the transport experiment (see Section 2.4). Images of one position were taken every 200 ms for 2 min in the CFP channel (excitation at 445 nm). The construct was chosen because of the good fluorescence properties of mTurquoise in the sensor [59].

The quantification of vesicle movement required a custom-made ImageJ macro. Background was subtracted using the rolling ball method and a bleaching correction based on an exponential fit was carried out on the 600 images per sample. The Otsu’s threshold clustering algorithm was applied, and particles sized 5–30 pixels, were defined as possible vesicles to allow tracking. Each cell was defined as a region of interest (ROI), and borders were drawn manually. We used the plugin TrackMate to get a readout of numerous parameters describing the movement of vesicles over time identifying MeanSpeed and MeanDisplacement as two of the most significant ones. While MeanSpeed describes the arithmetic mean of the mean speed of all captured vesicles in one ROI, MeanDisplacement is defined as the distance between the first position in a track and the last position in a track, providing a readout for directional and long-distance transport. The required parameters were drawn from the results files using a separate macro. 

### 2.6. Imaging and Analysis of the ER and Microtubule Network 

Investigations of the impact of energy stress on the morphology of the ER surface were conducted in HeLa cells expressing a truncated version (first 27 aa) of cytochrome P450 (CP450) C-terminally fused to circularly permutated venus (CPV), a yellow fluorescent construct via a flexible linker with the nucleotide sequence, i.e., AAACAGAAAGTGATGAACCAT. CP450-CPV localized at the surface of ER membranes. Before imaging, cells were treated for 3 × 10 min with measurement buffer containing no glucose (− Glu), 10 mM glucose (+ Glu, control), or 10 mM 2-DG (2-DG) as in ER-to-Golgi transport experiments (see Section 2.4), and then imaged in the respective buffer. For the analysis of microtubules, HeLa cells were transfected with GFP-α-tubulin on the day before the experiment. To avoid the collapse of the sensitive microtubule network before the imaging process, cells were directly transferred into a prewarmed storage buffer (see Section 2.1.). After transfer into a prewarmed measurement buffer containing 10 mM glucose or 10 mM 2-DG, respectively, the imaging process was immediately started using an array confocal laser scanning microscope (see Section 2.4.). For the image analysis, a custom-made ImageJ macro was applied. A mask isolating microtubules from the cytosolic signal was created by background subtraction in combination with median and FFT (fast Fourier transform) bandpass filter application. A threshold was set on the resulting image using the Otsu algorithm followed by opening and analysis of particles to create a mask defining the microtubule region. A ratio of mean intensities of microtubule regions and cytosolic regions was calculated to express any changes upon treatment for each position.

### 2.7. Calculation of Form Factor Changes in Fluorescent Structures 

The calculation of form factor values was conducted on the same set of images taken for the estimation of ER-to-Golgi transport in order to yield maximum comparability. A custom-made ImageJ macro was used to calculate the form factors. After background subtraction in the image showing the green fluorescent cargo construct, the Golgi region was removed based on the equivalent image from the RFP channel (showing Golgi marker mCherry-Golgi-7). Aggregates in the manually drawn region of interest were identified using a local threshold (Otsu) and the shape factor of all structures was calculated. Finally, the median form factor per cell was calculated for each time point.

### 2.8. Statistical Analysis

To ensure the comparability and reliability of statistical analyses, at least three independent experiments on at least three different days were performed in the data acquisition process. Cells of different passage numbers were analyzed. The numbers of individual tested cells per condition are indicated in the respective figure for each experiment. For the statistical analyses, we used Graphpad Prism software version 5.01 (GraphPad Software, San Diego, CA, USA). The results were tested for normal distribution using D’Agostino & Pearson omnibus normality test. For the comparison of the two groups, we used a t-test for normally distributed data, and the Mann–Whitney U test to analyze non-normally distributed data. The Kruskal–Wallis test for data not following a normal distribution and Dunn’s post hoc test for comparison of each dataset was applied for transport data. The comparison of frequency distributions was conducted via Chi-square test. *P*-values represented by asterisks are defined as < 0.05 (*, #), < 0.01 (**) and < 0.001 (***). 

## 3. Results

### 3.1. Single Cell Analysis Revealed Cargo-Dependent, Robust, and Efficient ER-to-Golgi Transport in HeLa Cells

**Figure 1:**
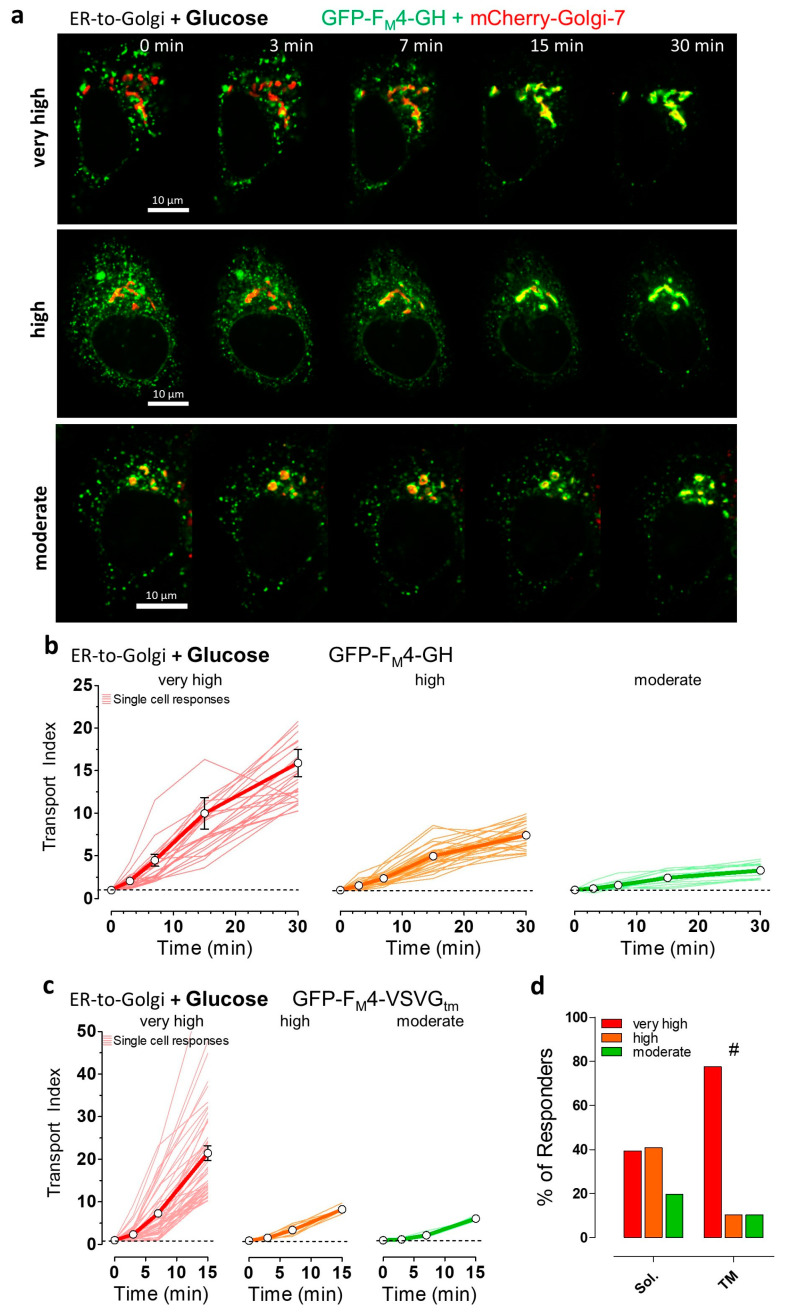
Visualization and classification of synchronized endoplasmic reticulum (ER)-to-Golgi transport in HeLa cells using soluble and transmembrane fluorescent protein (FP)-labeled cargos. (**a**) Representative images showing HeLa cells expressing GFP-F_M_4-GH, soluble cargo construct (in green), and mCherry-Golgi-7, Golgi marker (in red), and merged signals (in yellow) at the indicated time points upon the addition of 250 nM solubilizer in the presence of 10 mM glucose. ER-to-Golgi transport was classified as very high (upper panel), high (middle panel), and moderate (lower panel). A 100× magnification objective was used, scale bars indicate 10 µm in every image of the time course; (**b**) ER-to-Golgi transport of the soluble transport construct, GFP-F_M_4-GH in single HeLa cells (thin lines, n = 66 cells from 4 independent experiments) and respective mean ± SEM curves over time upon the addition of 250 nM solubilizer in the presence of 10 mM glucose. Transport efficiency was classified as very high (red curves, transport index > 10 at 30 min), high (orange curves, transport index > 5, <10 at 30 min), and moderate (green curves, transport index > 2, <5 at 30 min); (**c**) ER-to-Golgi transport of the transmembrane cargo construct (GFP-F_M_4-VSVG_tm_) under same conditions as described for panel (b). Classification of transmembrane cargo transport efficiency is based on the transport index after 15 min, i.e., very high transport (red curves, transport index > 10), high transport (orange curves, transport index > 7, <10), and moderate transport (green curves, transport index > 4, <7); (**d**) Bars showing the percentage of the different ER-to-Golgi transport efficiencies (very high, high, and moderate) of the soluble (Sol., left bars) and transmembrane (TM, right bars) cargo. Data are extracted from panels (b) and (c), respectively. # significant versus Sol. *p* < 0.05, Chi-square test.

To visualize and analyze synchronized ER-to-Golgi transport in individual living HeLa cells, we imaged fusion constructs that consist of conditional aggregation domains (CADs) fused to cargo proteins of the secretory pathway and a green fluorescent protein (GFP) variant. To study ER-to-Golgi transport of a classical bulk-flow cargo, a fusion construct consisting of an ER-targeting signal sequence, GFP, and CADs, as well as human growth hormone at the C-terminus, was imaged. To visualize the transport of a transmembrane cargo, the transmembrane domain of vesicular stomatitis virus G protein (VSVG) coupled to the C-terminus of GFP and CADs was used, working as an ER export signal, as well as a membrane anchor [41]. The co-expression of the green fluorescent cargo fusion construct with mCherry-Golgi-7, a red fluorescent marker of the Golgi complex, was used to co-localize the cargo construct with its primary target organelle, i.e., the Golgi apparatus (Figure 1a and Supplementary Figure S1). Before and immediately upon the addition of the solubilizer (D/D-Solubilizer, Takara), which binds to CADs inducing their disaggregation, the cargo fusion constructs appeared as green fluorescent puncta homogeneously distributed within the endoplasmic reticulum (ER) (Figure 1a and Supplementary Figure S1b). The amount of green fluorescent cargo co-localizing with mCherry-Golgi-7 prior to the addition of the solubilizer was very small in all HeLa cells tested. This subcellular distribution of the cargo fusion construct under basal conditions was expected, as the formation of protein aggregates by CADs is known to efficiently prevent cargo release from the ER towards the Golgi [52,53]. However, the addition of the solubilizer dissolved the green fluorescent puncta immediately and triggered synchronized ER-to-Golgi transport within minutes (Figure 1a and Supplementary Figure S1b). We could easily visualize the synchronized cargo transfer from the ER to the Golgi network in individual HeLa cells as an increase in the co-localization between green and red fluorescence and a decrease in green fluorescence in the ER, respectively, using high-resolution array confocal fluorescence microscopy (ACLSM).

To quantify ER-to-Golgi transport, a transport index based on green (i.e., cargo) and red (i.e., lumen of the Golgi complex) fluorescence co-localization was calculated at time points 3, 7, 15, and 30 min, respectively, after the addition of the solubilizer. To depict the heterogeneity of ER-to-Golgi transport, we arbitrarily classified all single responders into four groups based on differences in the automatically calculated transport index and subsequent optical control. Using human growth hormone (hGH) as a soluble, bulk-flow cargo in the fusion construct, we revealed that almost 40% of all HeLa cells showed a very fast and efficient ER-to-Golgi transport with a transport index >10 (“very high”, Figure 1a, upper panel, b left panel). In another 40% of cells, the same cargo was transported with a transport index between 5 and 10, which we classified as “high” ER-to-Golgi transport (Figure 1a middle panel, b middle panel). In the remaining 20% of analyzed HeLa cells, we observed ER-to-Golgi transport of the soluble cargo construct with a transport index between two and five, classified as “moderate” transport (Figure 1a lower panel, b right panel, and Supplementary Figure S1b left panel). Interestingly, we did not find any HeLa cells without any ER-to-Golgi transport activity of the soluble cargo construct upon the addition of the solubilizer. This arbitrary classification into four different groups of ER-to-Golgi transport efficiencies is based on the synchronized transport of this standard soluble cargo construct under control conditions on the level of single intact HeLa cells. We further used the same arbitrary classification of “very high”, “high”, “moderate”, and “no” transport of the bulk flow cargo, to compare ER-to-Golgi transport under different stresses.

Next, we analyzed ER-to-Golgi transport in single HeLa cells using vesicular stomatitis virus G protein (VSVG) transmembrane segment fused to the same luminal CADs and FP (Figure 1c and Supplementary Figure S1b right panel). In contrast to the luminal hGH construct, this construct contains a di-acidic ER export sequence displayed on the cytoplasmic surface of the ER, allowing active COPII sorting into vesicles. In line with our expectations and other reports [41,60], the transmembrane cargo construct was transported faster than the soluble cargo (Figure 1c). The ER-to-Golgi transport efficiency of the transmembrane cargo construct was also highly heterogeneous among individual HeLa cells. Thus, we again arbitrarily classified ER-to-Golgi transport of the transmembrane cargo into four groups under controlled conditions for further single-cell comparisons. In almost 80% of HeLa cells, the transmembrane construct showed a transport index >10, within 15 min after the addition of the solubilizer (“very high” transport activity, Figure 1c left panel, d). Among all HeLa cells tested, 10% of the cells were classified as showing either a “high” (transport index 7–10 within 15 min) or “moderate” (transport index 4–7 within 15 min) ER-to-Golgi transport of the transmembrane cargo construct (Figure 1c middle and right panels, d). To better assess the ER-to-Golgi transport activities of soluble and transmembrane constructs in HeLa cells, we additionally expressed the same constructs in NRK cells and visualized their transport. As compared to HeLa cells, similar transport efficiencies of both cargo constructs were observed in NRK cells, which represent a well-characterized cell model for high secretory transport efficiency [41,61,62] (Supplementary Figure S2). Interestingly, in contrast to HeLa cells (Figure 1), the mCherry-Golgi-7 construct expressed in NRK cells (Supplementary Figure S3a) stained several distributed small vesicular structures likely representing the trans-Golgi network. Thus, it was challenging to focus on the main Golgi apparatus before ER-to-Golgi transport of the fluorescent cargo was accomplished (Supplementary Figure S3b). These different characteristics of NRK cells yielded much smaller values for the transport index as compared with HeLa cells (Supplementary Figure S3c), despite similar ER-to-Golgi transport efficiency for both constructs in the kidney cells (Supplementary Figure S2a,b). In summary, these findings indicate that HeLa cells exhibit strong ER-to-Golgi transport activity and strong selectivity for COPII client cargoes.

### 3.2. Induction of Energy Stresses Characteristically Lowers Subcellular ATP Levels

**Figure 2:**
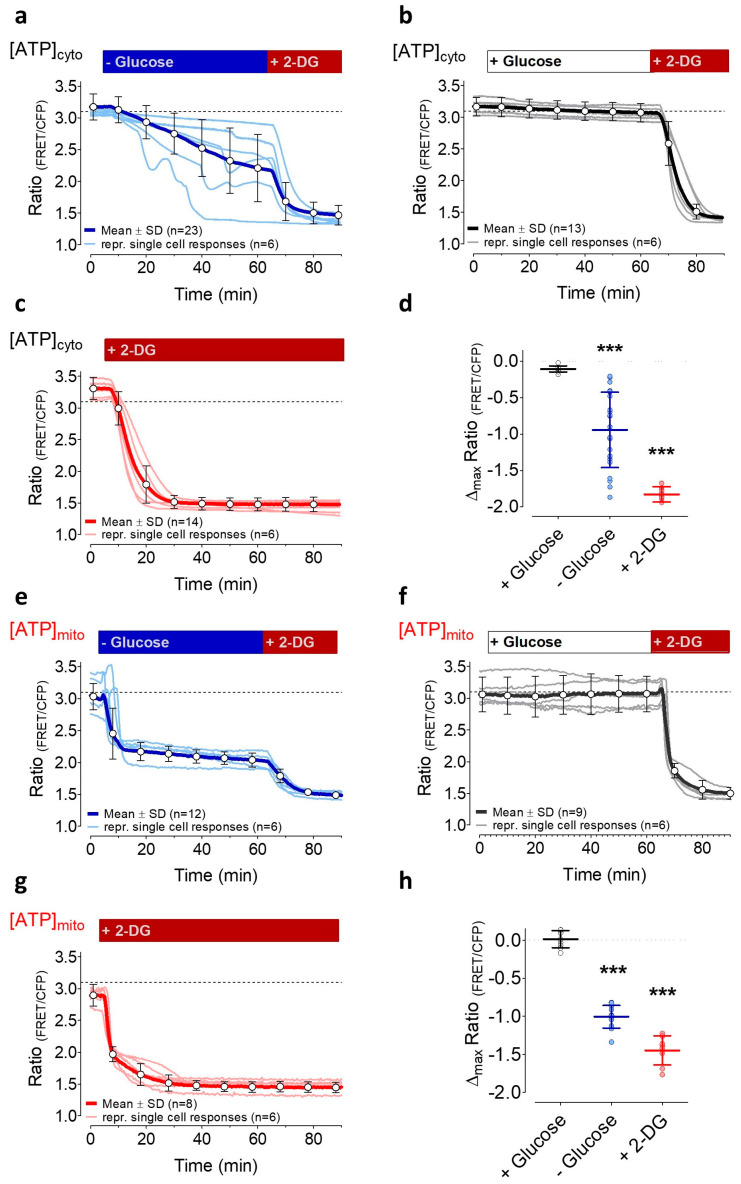
Cytosolic (**a**–**d**) and mitochondrial ATP (**e**–**h**) dynamics over time in response to energy stress. **(a**) Representative curves (light blue) and the mean response (± SD, blue curve, n = 23 cells from three independent experiments) of FRET ratio changes of cytosolic AT1.03 in HeLa cells over time upon the removal of 10 mM glucose and the subsequent addition of 10 mM 2-deoxy-D-glucose (2-DG), as indicated; (**b**) Representative FRET ratio changes (grey) and mean curve (± SD, black curve, n = 13 cells from three independent experiments) of cytosolic AT1.03 in HeLa cells in the presence of 10 mM glucose and upon the addition of 10 mM 2-DG; (**c**) FRET ratio changes (light red) and mean curve (± SD, red curve, n = 14 cells from three independent experiments) of cytosolic AT1.03 in HeLa cells that were treated with 10 mM 2-DG; (**d**) Aligned dot plot with mean of maximal FRET ratio changes of AT1.03 between basal values and at time point 60 min. *** significant versus control (+ glucose); unpaired *t*-test, *p* < 0.001. (**e**) Representative FRET ratio changes (light blue) and mean ratio changes (± SD, blue curve, n = 12 cells from three independent experiments) of mitochondria-targeted ATP-sensor mtAT1.03 upon glucose removal; (**f**) FRET ratio changes (grey) and mean response (± SD, black curve, n = 6 cells from three independent experiments) of mtAT1.03 over time in the presence of 10 mM glucose and the addition of 10 mM 2-DG; (**g**) Representative curves (light red) and mean ratio changes (± SD, red curve, n = 8 cells from three independent experiments) of mtAT1.03 upon replacement of glucose by 10 mM 2-DG, as indicated; (**h**) Aligned dot plot showing mean values of maximal FRET ratio changes of mtAT1.03 between basal values and values at time point 60 min. *** significant versus control (+ glucose), *p* < 0.001, unpaired *t*-test.

We exploited a well-established cytosolic Förster resonance energy transfer (FRET)-based ATP probe [47], AT1.03, coupled to a nuclear export sequence, to investigate the impact of energy stress on cytosolic ATP levels ([ATP]_cyto_) in HeLa cells for 60 min (Figure 2a–d). To our knowledge, high-resolution ATP imaging on the single-cell level under similar conditions has not yet been performed over such a time period. To mimic the common severe and persistent shortage of nutrient supply in a tumor, first, HeLa cells were rinsed for 60 min with an experimental salt buffer, which did not contain any sugars, amino acids, or any other metabolically usable substrates. Under these conditions, [ATP]_cyto_ in single HeLa cells declined very heterogeneously. Then, the subsequent addition of 2-DG maximally reduced [ATP]_cyto_ in all cells (Figure 2a). Interestingly, several cells showed oscillatory patterns of FRET ratio signals in the absence of glucose (Figure 2a), indicating that cytosolic ATP levels partially recover during the period of glucose starvation. However, after 60 min without glucose, the cytosolic ATP content in almost all cells was significantly reduced (Figure 2a,d). To exclude that other factors such as light exposure and shear stress in the perfusion chamber are responsible for the FRET ratio changes, analog experiments were performed in the continuous presence of glucose (Figure 2b). Under these conditions, FRET ratio signals stayed almost constant over time (Figure 2b,d), indicating that cytosolic ATP levels of HeLa cells remain high if cells are rinsed with a buffer containing high glucose. As expected, inhibition of glycolysis by exchanging glucose with 2-DG in the same set of experiments lowered [ATP]_cyto_ to a minimum within several minutes (Figure 2b). To investigate whether [ATP]_cyto_ remained low over time when glycolysis is inhibited by 2-DG, additional experiments were performed in the continuous presence of 2-DG (Figure 2c). The addition of 2-DG rapidly reduced [ATP]_cyto_ and cytosolic ATP levels remained constantly low over 60 min. These experiments demonstrated that the removal of glucose and the addition of 2-DG lowered [ATP]_cyto_ in HeLa cells with different potency and dynamic. 

Since mitochondria and the mitochondrial ATP pool represent a dynamic hub of the cell’s energy homeostasis [63], we next measured the impact of long-term glucose starvation on [ATP]_mito_ using the genetically encoded FRET sensor mtAT1.03, which is targeted to mitochondria [47]. Despite the high heterogeneity in cytosolic ATP (Figure 2a,d) under glucose starvation, HeLa cells reacted rather homogenously with a brief transient increase followed by a sharp drop in mitochondrial ATP, which remained constantly low in the absence of glucose (Figure 2e). However, the addition of 2-DG further decreased [ATP]_mito_ to a minimum, suggesting that ATP consumption by the glucose antimetabolite additionally lowers the ATP content of the organelle. In the presence of glucose, cells stably retained [ATP]_mito_ for 60 min (Figure 2f). The subsequent exchange of glucose by 2-DG, however, induced a sharp drop of [ATP]_mito_ within several minutes (Figure 2f). In line with this observation, cell treatment with 2-DG throughout the experiment also instantly reduced [ATP]_mito_ to its minimum (Figure 2g). In the presence of 2-DG, [ATP]_mito_ remained at minimal levels for 60 min (Figure 2g). These findings showed that ATP levels in the cytosol and mitochondria are affected differently depending on the type of energy stress induction. Moreover, these protocols establish conditions of moderate and severe subcellular ATP depletion, which allow further assessment of the energy dependency and energy-stress sensitivity of ER-to-Golgi transport. 

### 3.3. Partial ATP Depletion by Glucose Deprivation Enhances ER-to-Golgi Transport but Complete ATP Depletion Using 2-DG Prevents this Transport 

**Figure 3:**
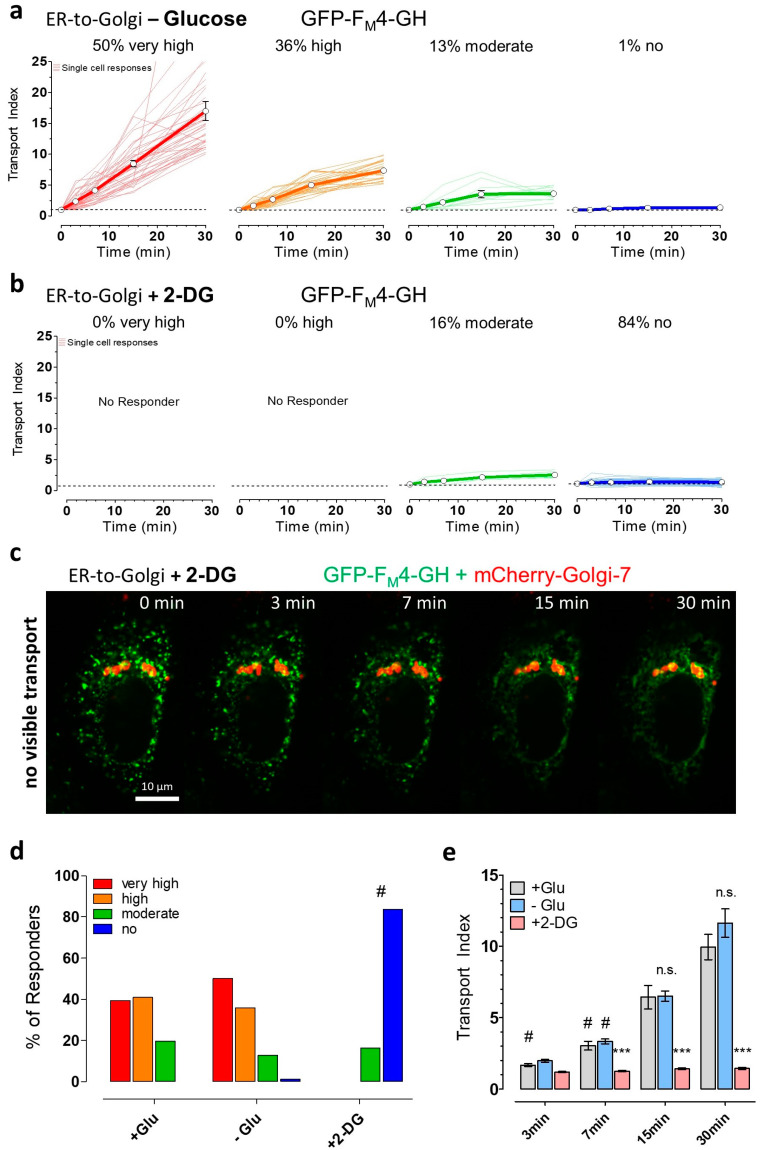
ER-to-Golgi transport of soluble cargo is facilitated by short-term glucose starvation but impaired by 2-DG. (**a**) ER-to-Golgi transport of GFP-F_M_4-GH in glucose-deprived single HeLa cells (n = 78 cells from 3 independent experiments) showing very high (red, transport index at 30 min > 10), high (orange, transport index > 5, <10), moderate transport index (green, transport index > 2, <5), and no transport (blue, transport index < 2). Cells were treated with buffer without glucose for 30 min prior to transport initiation by the addition of 250 nM solubilizer at 37 °C; (**b**) Classification as described in panel (a) of ER-to-Golgi transport in HeLa cells that were treated with 10 mM 2-DG, during and before (30 min) the addition of 250 nM solubilizer (n = 61 cells from 4 independent experiments); (**c**) A representative time course showing a 2-DG treated HeLa cell lacking ER-to-Golgi transport over time. Images were taken directly before solubilizer-addition (0 min) and 3, 7, 15, and 30 min after the addition of 250 nM solubilizer, respectively. The images show an overlay of the green channel (GFP-F_M_4-GH, soluble cargo) and red channel (mCherry-Golgi-7, Golgi marker). A 100× magnification objective was used, scale bar indicates 10 µm for every image in the time course; (**d**) Bars show the percentages of different ER-to-Golgi transport efficiencies in presence of 10 mM glucose (left bars, + Glu, control, n = 66 cells from 4 independent experiments), the absence of glucose (middle bars, − Glu, n = 78 cells from 3 independent experiments) and upon cell treatment with 10 mM 2-DG (right bars, + 2-DG, n = 61 cells from 4 independent experiments). # significant versus + Glu *p* < 0.05, Chi-square test; (**e**) Mean transport index comparison of HeLa cells of different pretreatment conditions (+ Glu (grey), n = 66 cells, − Glu (blue), n = 78 cells, + 2-DG (red), n = 61 cells) at the indicated time points after transport initiation. Bars represent mean ± SEM. n.s. not significant versus control (+ Glu) at the same time point, *** significant versus control (+ Glu) at the same time point *p* < 0.001, # significant versus all other time points of the same condition *p* < 0.05, Kruskal–Wallis test and Dunn’s post hoc test.

To further investigate the impact of different energy stresses on ER-to-Golgi transport, a three-step protocol was established (Supplementary Figure S1a). In the first step, HeLa cells were co-transfected with the cargo-fusion construct and the Golgi marker mCherry-Golgi-7 under resting control conditions (see Section 3.1., Figure 1). To induce different levels of energy stress, cells were additionally either starved by the removal of glucose or the addition of 2-DG for 30 min before imaging experiments and the addition of the solubilizer. In Supplementary Figure S1a, the experimental setup is depicted to allow a correlation of subcellular ATP changes with ER-to-Golgi transport. Although glucose starvation strongly reduced ATP levels within mitochondria and partially lowered the cytosolic ATP content (Figure 2), the removal of glucose did not reduce ER-to-Golgi transport of the soluble cargo (GFP-F_M_4-GH, Figure 3a). Surprisingly, based on our arbitrary classification, glucose starvation increased the number of cells which showed very high ER-to-Golgi transport by roughly 10% (Figure 3a,d) as compared with the control conditions. Consequently, single HeLa cells, with low or moderate ER-to-Golgi transport, were less frequently found (Figure 3a,d), indicating that glucose removal, despite clear subcellular ATP reductions, as well as fluctuations (Figure 2), does not reduce ER-to-Golgi transport (Supplementary Figure S4), but might even facilitate the transfer of bulk-flow secretory cargo from the ER towards the Golgi apparatus. Notably, this effect of a slightly increased ER-to-Golgi transport of the soluble cargo in the absence of glucose was observed only when analyzing the transport index of individual cells. By analyzing the cell population, we were only able to confirm that glucose starvation did not significantly reduce ER-to-Golgi transport of the bulk-flow cargo (Figure 3e). In contrast to glucose starvation, HeLa cell treatment with 2-DG abolished visible ER-to-Golgi transport of the luminal construct (Figure 3b–d) in the majority of cells; moderate ER-to-Golgi transport was still observable but only in a very small fraction of HeLa cells (Figure 3b,d). Under these conditions of strong subcellular ATP depletion (Figure 2), we did not observe any visible effects on the ER surface morphology (Supplementary Figure S5) or on the nocodazole-sensitive cellular microtubule network (Supplementary Figure S6). To investigate the impact of severe energy stress on the morphology and dynamics of microtubules in intact living cells, we expressed GFP-α-tubulin and performed high-resolution fluorescence imaging. Interestingly, we could observe microtubule remodeling in the controls and also in the presence of 2-DG, as well as disaggregation upon the addition of nocodazole (Supplementary Figure S6). HeLa cells also showed clear disaggregation of green fluorescent cargo upon the addition of solubilizer in the presence of 2-DG (Supplementary Figure S7a). These observations indicate that the lack of significant cargo co-localization with the Golgi marker (Figure 3c) was due to a block in transport, per se, as opposed to more general morphological disruptions. 

To further investigate the impact of energy stress on the ability of the solubilizer to disaggregate the cargo fusion construct, the morphology changes of ER-localized puncta of the soluble constructs were analyzed over time. In the presence of glucose, the addition of solubilizer induced a significant increase in the form factor of green fluorescent structures within 7 min, indicating the efficient disaggregation of the ER-located puncta into the fine ER network (Supplementary Figure S7a). Cell treatment with 2-DG did not prevent a significant increase in the form factor of green fluorescent cargo construct, indicating that the disaggregating effect of the solubilizer remains high under these energy stress conditions. As compared with the control conditions, the form factor of green subcellular structures increased more noticeably in the presence of 2-DG (Supplementary Figure S7b), likely due to the inhibition of cargo transport into small vesicular structures, which would yield low form factors.

**Figure 4:**
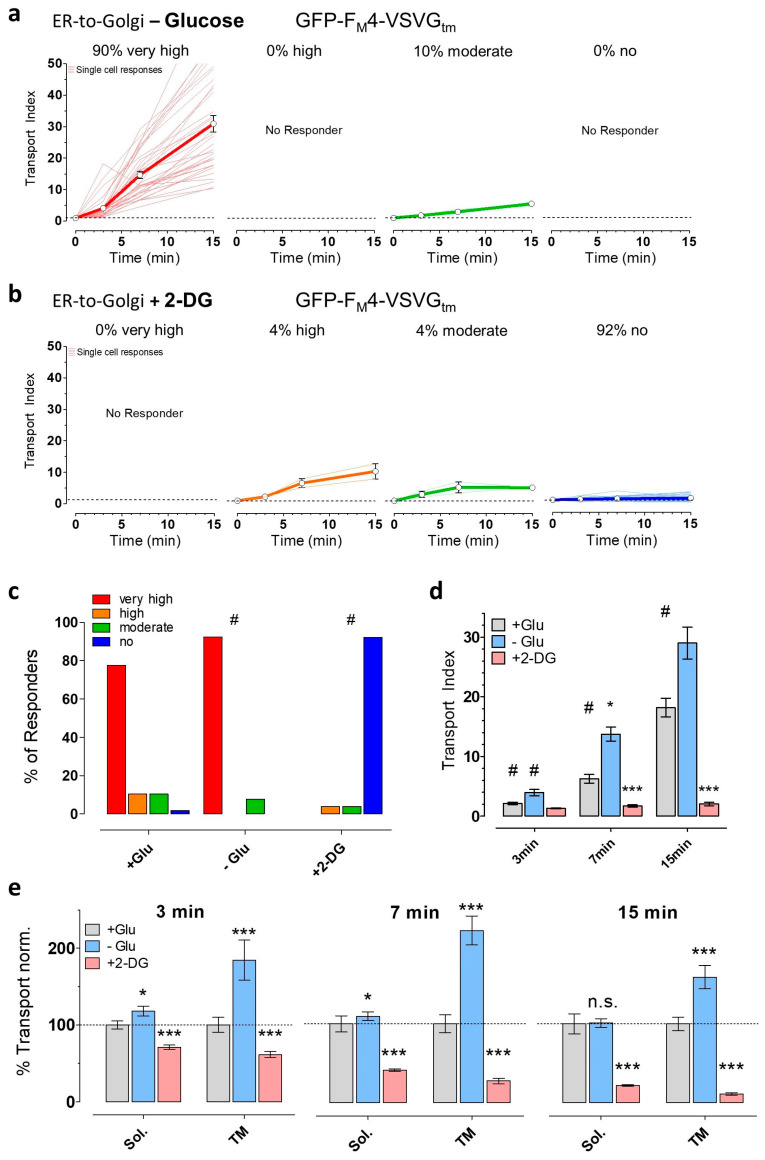
ER-to-Golgi transport of the transmembrane cargo construct in HeLa cells is boosted by glucose starvation but blocked by 2-DG. (**a**) Classification of glucose-deprived HeLa cells (n = 39 cells from 3 independent experiments) based on the efficiency of ER-to-Golgi transport of GFP-F_M_4-VSVG_tm_. Cells were treated with buffer lacking glucose for 30 min prior to transport initiation. Transport was classified as very high (red, transport index at 15 min > 10), high (orange, transport index > 7, <10), moderate (green, transport index > 4, <7), or no transport (blue, transport index < 4); (**b**) Classification of 2-DG-treated HeLa cells (n = 51 cells from 4 independent experiments) based on ER-to-Golgi transport efficiency. Transport was initiated and measured after pretreating cells with buffer containing 10 mM 2-DG for 30 min before the addition of solubilizer; (**c**) Comparison of the percentages of tested HeLa cells in the four classes for each pretreatment condition. Cells were treated for 30 min before transport initiation with buffer containing 10 mM glucose (+ Glu, control, n = 58 cells from 4 independent experiments), no glucose (− Glu, n = 39 cells from 3 independent experiments), or 10 mM 2-DG (+ 2-DG, n = 51 cells from 4 independent experiments). # significant versus + Glu *p* < 0.05, Chi-square test; (**d**) Transport index comparison of HeLa cells of different pretreatment conditions (+ Glu (grey), n = 58 cells, − Glu (blue), n = 39 cells, + 2-DG (red), n = 51 cells) at the indicated time points after transport initiation. Bars represent mean ± SEM. *** significant versus control (+ Glu) at the same time point *p* < 0.001, # significant versus all other time points of the same condition *p* < 0.05, Kruskal–Wallis test and Dunn’s post hoc test; (**e**) Transport of soluble (Sol., GFP-F_M_4-GH) and transmembrane construct (TM, GFP-F_M_4-VSVG_tm_) normalized to control conditions at indicated time points. Bars represent mean values ± SEM. * significant versus control (+ Glu) at the same condition *p* < 0.05, *** *p* < 0.001, Mann–Whitney U test.

Next, we investigated if the inductions of energy stress also affected the transport of the transmembrane domain of vesicular stomatitis virus G protein (VSVG). In contrast to GFP-F_M_4-GH, which represents a luminal cargo transported via bulk flow, GFP-F_M_4-VSVG_tm_ is actively sorted into COPII vesicles due to its cytoplasmic ER export sequence [64], allowing more efficient transport (Figure 1d). Interestingly, despite these very different transport mechanisms for the two constructs, glucose deprivation also significantly increased the synchronized ER-to-Golgi transport of the transmembrane construct (Figure 4a,c). Using our arbitrary classification of different ER-to-Golgi transport efficiencies, we again observed 10% more cells with a very high transport index of the transmembrane construct if cells were starved. The addition of 2-DG, however, completely prevented visible transport of the transmembrane construct in over 90% of tested HeLa cells (Figure 4b,c).

Furthermore, we compared the mean transport index values at every time point for each treatment condition (Figure 3e and Figure 4d). These analyses additionally confirmed that the transmembrane construct was better transported in glucose-deprived cells as compared with the control conditions, while 2-DG strongly inhibited ER-to-Golgi transport under these conditions (Figure 4d). Notably, the analogous data for the soluble construct did not show a significant difference between + Glu and – Glu at time point 30 min (Figure 3e), despite the observed increase in cells showing very high ER-to-Golgi transport (Figure 3a), which highlights the additional value of a single cell analysis approach in addition to the comparison of ER-to-Golgi transport efficiencies of whole-cell populations. Although the trend of effects of glucose withdrawal was similar for both cargoes, the transport of transmembrane cargo was influenced stronger by the absence of glucose than was the soluble, bulk flow cargo (Figure 4e). The potentiation of transport by glucose withdrawal may, thus, affect cargo sorting as opposed to just vesicle formation or other steps. In addition, both cargoes underwent a similarly striking inhibition of transport by 2-DG, perhaps indicating a broader effect on multiple aspects of transport. 

At 30 min after the addition of the solubilizer, some cells showed reduced levels of the GFP signal of the transmembrane construct within the bulk Golgi apparatus (Supplementary Figure S8a), showing that the construct is rapidly further transported. Interestingly, this effect was again pronounced in glucose-deprived cells (Supplementary Figure S8b), confirming accelerated secretory transport under these energy stress conditions.

### 3.4. The Induction of Energy Stress Strikingly Alters Cellular Ca^2+^ Homeostasis and Mobilization, and Ca^2+^ Signals Differentially Impact ER-to-Golgi Transport as Compared with 2-DG 

**Figure 5:**
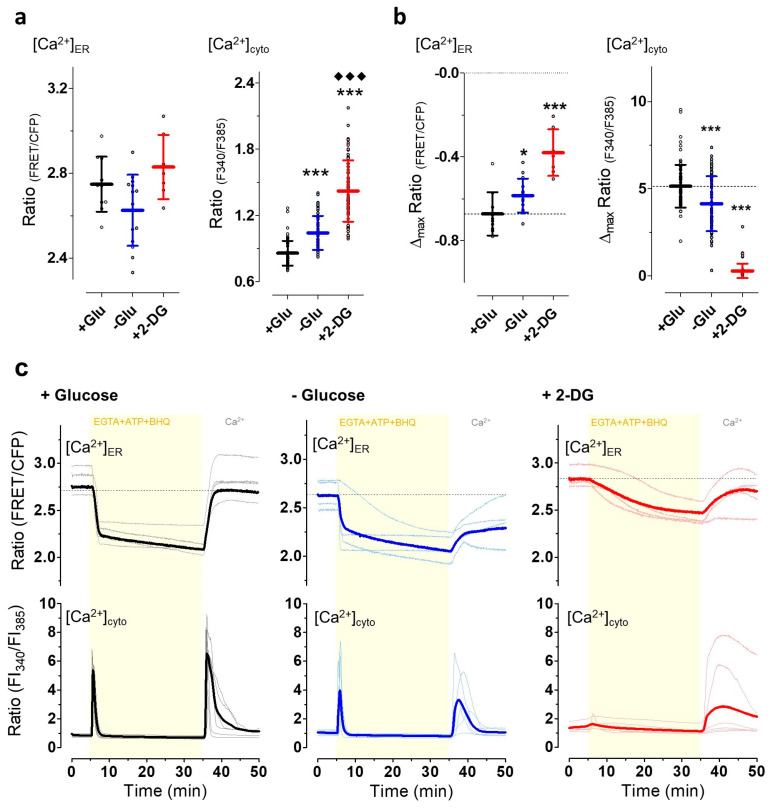
Mild and severe energy depletion affects the cytosolic and ER Ca^2+^ homeostasis. For measurement of cytosolic calcium dynamics HeLa cells were loaded with Fura-2 (+ Glu, n = 78 cells from four independent experiments; − Glu, n = 84 cells from three independent experiments; + 2-DG n = 73 cells from four independent experiments), for visualizing ER-calcium HeLa cells expressing D1ER were measured (+ Glu, n = 10 cells; − Glu, n = 14 cells; and + 2-DG n = 7 cells; each from three independent experiments). (**a**) Aligned dot plot and mean ± SD representing FRET ratio values of D1ER (i.e., [Ca^2+^]_ER_, left) and Fura-2 ratio values (i.e., [Ca^2+^]_cyto_, right) of unstimulated HeLa cells that were kept in the presence of 10 mM glucose (+ Glu, black), upon glucose starvation for 30 min (− Glu, blue), or cell treatment with 10 mM 2-DG for 30 min (+ 2-DG, red). *** significant versus + Glu *p* < 0.001, ♦♦♦ significant versus − Glu *p* < 0.001, Mann–Whitney U test; (**b**) Aligned dot plot and means ± SD showing the maximum change in ratio signals of D1ER (left) and Fura-2 (right) upon cell stimulation with ATP (100 µM) and 2,5-di-t-butyl- 1,4-benzohydroquinone (BHQ) (15 µM) in the absence of extracellular Ca^2+^ (i.e., EGTA), as also shown in panel c. * significant versus + Glu *p* < 0.05, *** significant versus + Glu *p* < 0.001, Mann–Whitney U test; (**c**) ER (upper panel) and cytosolic (lower panel) Ca^2+^ signals over time in the presence of 10 mM glucose (+ Glu control, black, left panels, n_ER_ = 10 cells, n_cyto_ = 78 cells), the absence of glucose (− Glu, blue, middle panels, n_ER_ = 14 cells, n_cyto_ = 84 cells), and upon cell treatment with 10 mM 2-DG (30 min prior to imaging experiments, + 2-DG red, right panels, n_ER_ = 7 cells, n_cyto_ = 73 cells). As indicated, cells were stimulated with a mixture of 100 µM ATP and 15 µM BHQ in the absence of extracellular Ca^2+^, i.e., in the presence of 0.1 mM EGTA. Subsequently, ATP and BHQ were removed and 2 mM Ca^2+^ was re-added via a perfusion system.

It has been suggested that energy stresses with reductions in the subcellular ATP content might consequently also affect cellular Ca^2+^ homeostasis [45]. Thus, we measured the Ca^2+^ levels and dynamics of the ER and cytosol, respectively, under conditions of energy stress (Figure 5). For ER Ca^2+^ measurements in HeLa cells, we expressed D1ER, a well-established genetically encoded FRET-based Ca^2+^ biosensor targeted to the ER [65]. We initially expected that ER Ca^2+^ storage might be reduced under conditions of energy stress due to the clear ATP dependency of the (sarco)endoplasmatic Ca^2+^ ATPase (SERCA) [66]. However, neither glucose removal nor exchanging glucose by 2-DG significantly reduced the basal D1ER ratio values, indicating that the ER Ca^2+^ content was not lowered under these conditions of moderate and severe energy stress (Figure 5a left). In parallel experiments, Fura-2 was used to estimate the impact of energy stresses on cytosolic Ca^2+^. Under conditions of energy stress by glucose deprivation, the basal Fura-2 ratio values were significantly increased, indicating elevated cytosolic Ca^2+^ levels in HeLa cells. The effect on the basal Fura-2 ratio values was more pronounced when glucose was exchanged by 2-DG, indicating that the strong reduction in ATP was accompanied by a remarkable elevation of cytosolic Ca^2+^ levels (Figure 5a, right). 

Next, we tested how energy stress impacts Ca^2+^ signals in HeLa cells. For this purpose, we triggered maximal inositol 1,4,5-triphosphate (IP_3_)-dependent Ca^2+^ release from the ER by cell treatment with the IP_3_-generating agonist ATP in combination with 2,5-di-tert-butylhydroquinone (BHQ), which despite potential side effects in cardiomyocytes [67], works as a specific SERCA inhibitor in non-excitable cells, in the absence of extracellular Ca^2+^ and upon Ca^2+^ re-addition in the absence of these chemicals. Under control conditions in the presence of glucose, first, this treatment lowered the ER Ca^2+^ content (Figure 5b left, c upper panel) rapidly and, consequently, triggered a transient cytosolic Ca^2+^ signal (Figure 5b right, c lower panel). The removal of the IP_3_-generating agonist together with the SERCA inhibitor upon re-addition of Ca^2+^ to the extracellular medium, completely restored ER Ca^2+^ within minutes and transiently elevated cytosolic Ca^2+^ levels under control conditions. Ca^2+^ mobilization by ATP and BHQ was less effective and more heterogeneous if cells were stressed by glucose deprivation (Figure 5c). Moreover, in the absence of glucose, ER Ca^2+^ refilling and respective cytosolic Ca^2+^ transients were also reduced (Figure 5c). Under conditions of severe energy stress induced by cell treatment with 2-DG, Ca^2+^ mobilization from the ER by ATP and BHQ occurred slowly and less pronounced with only small, hardly detectable cytosolic Ca^2+^ changes. However, the re-addition of Ca^2+^ along with the removal of ATP and BHQ sometimes triggered quite strong cytosolic Ca^2+^ elevations, which were accompanied by a slow and inefficient ER Ca^2+^ refilling (Figure 5c, upper panel).

**Figure 6:**
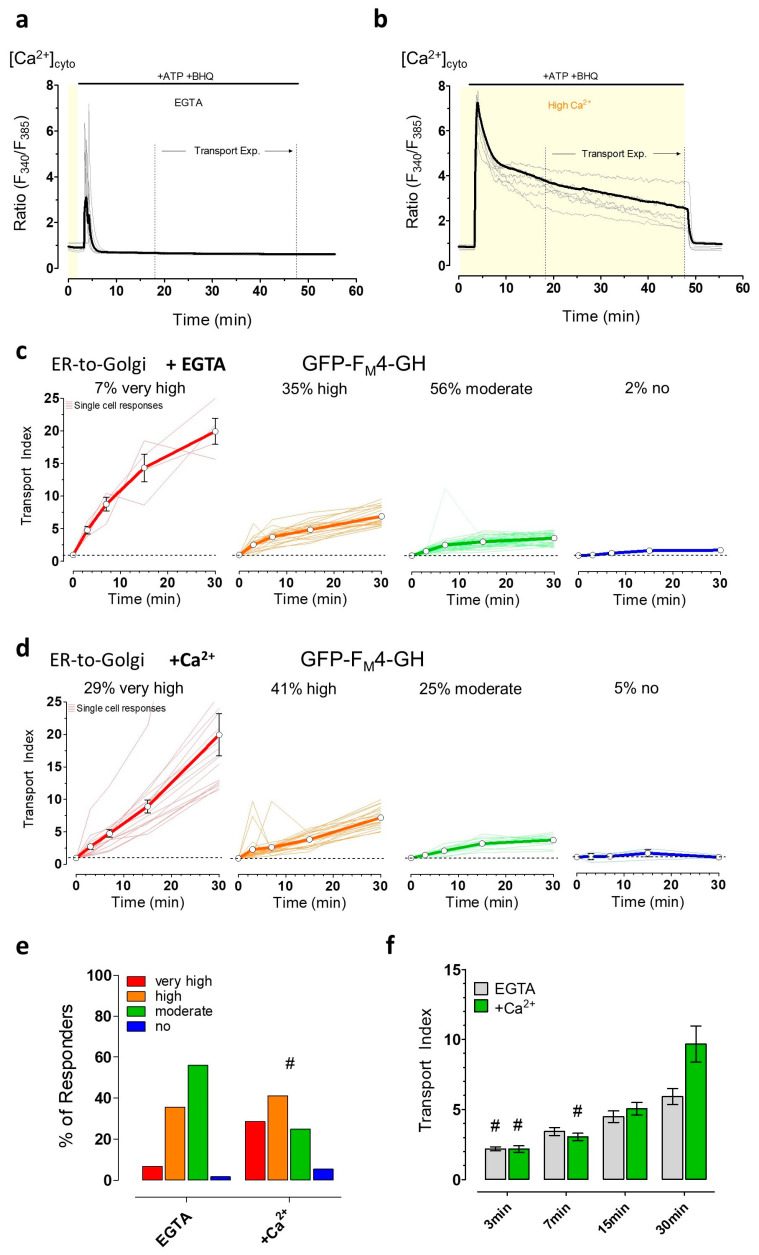
ER-to-Golgi transport of soluble cargo is affected by short-term calcium mobilization in HeLa cells. (**a**,**b**) Curves showing changes in Fura-2 ratio (i.e., cytosolic Ca^2+^ concentrations) over time upon Ca^2+^ mobilization using 100 µM ATP and 15 µM BHQ in the absence of extracellular Ca^2+^ (i.e., 0.1 mM EGTA, n = 53 cells from three independent experiments) (a) and presence of 2 mM Ca^2+^ (n = 66 cells from three independent experiments) (b). The respective time frame after Ca^2+^ mobilization for transport experiments (**c**–**f**) is indicated with dotted lines (“Transport Exp.“). (c) Curves represent very high, high, moderate, and no ER-to-Golgi transport of GFP-F_M_4-GH in single HeLa cells (n = 59 cells from 3 independent experiments) after 15 min of Ca^2+^-mobilization in EGTA; (d) ER-to-Golgi transport in single HeLa cells (n = 56 cells from three independent experiments) under conditions of Ca^2+^ mobilization in the presence of Ca^2+^, as shown in panel b; (e) Bars represent the percentages of different transport efficiencies of the soluble cargo of HeLa cells treated with ATP and BHQ in the absence (i.e., EGTA) or presence (+ 2 mM Ca^2+^) of extracellular Ca^2+^; (f) Comparison of mean transport index ± SEM in stimulated HeLa cells (100 µM ATP plus 15 µM BHQ) in EGTA (grey bars, n = 59 cells,) or in the presence of 2 mM extracellular Ca^2+^ (green bars, n = 56 cells) at the indicated time points of transport. # significant versus all other time points of the same condition *p* < 0.05, Kruskal–Wallis test and Dunn’s post hoc test.

These clear effects of energy stress on the subcellular Ca^2+^ handling prompted us to further investigate the possible impacts of ER Ca^2+^ mobilization and specifically cytosolic Ca^2+^ signals on ER-to-Golgi transport. Thus, we depleted ER Ca^2+^ by ATP and BHQ either in the absence (Figure 6a) or presence of extracellular Ca^2+^ (Figure 6b) which allowed us to investigate the impact of ER Ca^2+^ depletion and elevated cytosolic Ca^2+^ on ER-to-Golgi transport. In the absence of extracellular Ca^2+^, cytosolic Ca^2+^ transiently increased, and then remained lower than basal cytosolic Ca^2+^ levels during cell stimulation (Figure 6a). In the continuous presence of extracellular Ca^2+^, however, elevated cytosolic Ca^2+^ levels remained high during the entire time of cell stimulation (Figure 6b). Synchronized ER-to-Golgi transport of the luminal green fluorescent cargo construct could be observed in almost all cells upon cell stimulation with ATP and BHQ in the absence (Figure 6c) and presence of extracellular Ca^2+^ (Figure 6d). Nevertheless, in the absence of extracellular Ca^2+^, as compared with in the presence of extracellular Ca^2+^, the number of cells with a very high transport index of >10 was reduced, while that of a moderate transport rate was increased. The number of HeLa cells with a high transport index of ER-to-Golgi transport was similar for both conditions (Figure 6e). A comparison of mean transport index values of the cell population at each time point showed no significant differences after 3, 7, and 15 min and yet a slightly higher transport index at 30 min for cells treated in the presence of extracellular Ca^2+^ (Figure 6f). 

### 3.5. The Induction of Energy Stress by 2-DG and Cytosolic Ca^2+^ Elevations both Efficiently Impair Vesicle Movements

**Figure 7:**
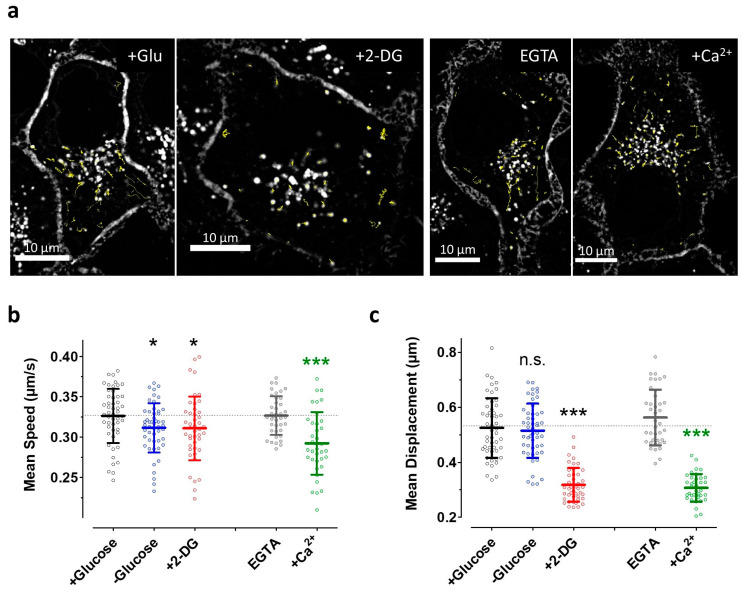
Vesicle motility in HeLa cells is differentially affected by Ca^2+^ and energy stresses. (**a**) Confocal images of HeLa cells stably expressing pH-Lemon-GPI that were used for time-course vesicle movement experiments. The first images in the time courses of representative cells of different treatment conditions (+ Glu and + 2-DG, left panel; EGTA and + Ca^2+^, right panel) are shown. The tracks of all detected moving vesicles in 600 images over 2 min for each individual cell are shown in yellow. A 100× magnification objective was used, scale bars represent 10 µm; (**b**,**c**) Scatter dot plots and mean values ± SD representing mean speed (b) and mean displacement (c) for each cell measured at different (pre-)treatment conditions, i.e., + 10 mM glucose without any cell stimulation (control, black, n = 51 cells), − glucose (blue, n = 44), + 10 mM 2-DG (+ 2-DG, red, n = 50), cell treatment with 100 µM ATP and 15 µM BHQ in EGTA (grey, n = 39), or 2 mM extracellular Ca^2+^ (+ Ca^2+^, green, n = 37). n.s. Not significant versus + glucose (control), * significant versus + glucose (control) *p* < 0.05, *** significant versus + glucose (control) *p* < 0.001, *** (green) significant versus EGTA *p* < 0.001, unpaired *t*-test.

We exploited a genetically encoded glycosylphosphatidylinositol (GPI)-anchored fluorescent construct, the pH-Lemon-GPI, to investigate the impact of energy and Ca^2+^ stresses on the overall motility of vesicular structures. While these experiments might be less conclusive regarding the motility of ER-to-Golgi intermediates, they allow a good estimation of how Ca^2+^ mobilization and ATP depletion acutely affect the movement of GPI-positive transport intermediates more generally (in part dependent on motor proteins). Thus, these data represent an additional readout to compare the sensitivity of general vesicle dynamics and ER-to-Golgi transport to the same Ca^2+^ and energy stresses in a cancer cell line. High-frequency confocal imaging at five images per second revealed strikingly reduced vesicle dynamics in HeLa cells under energy or Ca^2+^ stress as compared with controls (Supplementary Videos S1–S4); this is also made clear by looking at individual vesicle tracks in single HeLa cells (Figure 7a). Custom-made algorithms for semi-automated image analysis also allowed the quantification of these movements (Figure 7b,c). Glucose starvation for 30 min slightly reduced the average speed of single vesicles within HeLa cells (Figure 7b, left two lanes), while long-distance movements were not significantly affected under these conditions (Figure 7c, left two lanes). The induction of severe energy stress by 2-DG also only slightly reduced the average velocity of vesicle transport (Figure 7b, lanes 1 vs. 3) but strongly impaired long-distance movements of pH-Lemon-GPI-positive vesicles (Figure 7c, lanes 1 vs. 3). Cell treatment with the Ca^2+^ mobilizing combination of the IP_3_, generating ATP and SERCA inhibitor BHQ in the absence of extracellular Ca^2+^ (i.e., in the presence of EGTA), on the one hand, did not affect the average speed or the long-distance movements of vesicles in HeLa cells (Figure 7a–c and Supplementary Video S3). On the other hand, in the presence of extracellular Ca^2+^, which yielded elevated cytosolic Ca^2+^ levels upon cell treatment with ATP, and BHQ (Figure 6) strongly impaired vesicle speed and motility (Figure 7b,c, lanes 4 vs. 5). These data indicate that conditions of strongly reduced vesicle movements, such as those induced by, for example, cytosolic Ca^2+^ elevations, do not necessarily correlate with an impaired ER-to-Golgi transport. However, severe energy stress by 2-DG reduced both vesicle movements and ER-to-Golgi transport.

## 4. Discussion

In this in vitro study, we used HeLa cells and mimicked short-term energy stress conditions by either removing glucose from the medium or by exchanging it with 2-DG. We assessed the effect of these energy stress conditions on individual cancer cells by imaging subcellular ATP and Ca^2+^ dynamics exploiting well-established genetically encoded fluorescent biosensors. Moreover, the main focus of this study was on the quantitative visualization of the impact of different short-term energy stresses on ER-to-Golgi transport, which represented an initial and central step of protein secretion [1]. Exploiting GFP technology for quantifying ER-to-Golgi transport in live HeLa cells, our study is informative regarding the actual influence of the metabolic activity on the secretory pathway in a well-known cancer cell model.

It has been well documented that cancer cells have a high capacity to secrete proteins, as well as other biomolecules [3]. The secretory pathway in cancer cells is believed to contribute to cancer cell biology by facilitating interaction with the extracellular matrix and also with other cells including immune cells and cells of the vasculature within the tumor microenvironment [1,3,68,69,70]. Our comparison of single-cell ER-to-Golgi transport between NRK cells, a kidney cell model with high secretion rates commonly used to study the secretory pathway [41], and HeLa cells indeed confirmed similarly strong secretory activity in the cancer cell model. Cellular secretion of biomolecules controls cancer cell viability, growth, and metastasis [3]. Given the central role of protein secretion in cancer cells, targeting the secretory pathway seems promising for the design of novel strategies in the fight against cancer. While some specific compounds that inhibit the secretory activity [15] such as Brefeldin A [71], indeed exhibit clear antiproliferative effects [15,72,73,74], such drugs, so far, have not been commonly used in cancer therapy [15]. However, it is tempting to speculate that several anticancer drugs are at least partially effective via affecting the secretory activity of cancer cells. Given the essential requirement of energy supply for maintaining secretion in cells, anti-metabolic drugs might also effectively disturb the secretory activity of cancer cells. While our findings using 2-DG confirmed the high energy dependency of the secretory pathway, as ER-to-Golgi transport was almost completely abolished in the presence of the glucose analog, the removal of glucose alone seemed to facilitate ER-to-Golgi transport, despite clear global cytosolic and mitochondrial ATP reductions under these conditions. These effects were not restricted to luminal cargo transported via bulk flow but could also be visualized, to an even greater degree, with cargo that was actively and efficiently sorted into vesicles. While the analysis of single-cell ER-to-Golgi transport and the arbitrary classification into different groups under control conditions and upon cell stress should be interpreted with caution, we speculate that this phenomenon of facilitating ER-to-Golgi transport by glucose deprivation might be based on cancer cell-specific metabolic setting. Cancer cells of fast-growing tumors are metabolically flexible to cope with fluctuations in substrate and oxygen supply [75]. Many such tumor cells switch to aerobic glycolysis, a metabolic setting well-known as the Warburg effect [54,55], which facilitates glucose uptake and increases rates of optimized glycolysis for the fast generation of ATP as well as metabolic building blocks [56]. We and others recently demonstrated that HeLa cells relied on mitochondria-localized hexokinase 1 and 2 (HK-1, HK-2), which were fueled by mitochondrial ATP to efficiently phosphorylate glucose [40,76]. Interestingly, it seems that this metabolic setting requires ATP import into mitochondria from glycolysis in the cytosol to maintain the metabolic activity of HeLa cells. Glucose removal was shown to even initially elevate the mitochondrial ATP pool under these conditions [40]. Thus, we further speculate that under conditions of energy stress, which is induced by glucose removal, initially even more ATP or GTP might be available locally for ER-to-Golgi transport. It is unknown why the supportive effect of glucose deprivation on ER-to-Golgi transport was stronger for an actively sorted cargo than for the bulk flow cargo. One speculation would be that since COPII cargo sorting requires multiple rounds of GTP hydrolysis by Sar1 [77], the transport of actively-sorted COPII cargo may be more energy-limited than that of bulk flow cargo.

In contrast to glucose removal, the addition of 2-DG strongly prevented ER-to-Golgi transport in almost all HeLa cells. This observation is in line with a clear and homogeneous reduction in 2-DG on cytosolic, as well as mitochondrial ATP levels. Interestingly, glucose removal induced much slower and sometimes oscillatory ATP reductions in the cytosol of HeLa cells, although ATP biosynthesis in this cancer cell types heavily relies on glycolysis [40]. We think that high concentrations of glycolysis intermediates, ATP supply from the mitochondrial pool, as well as energy stress pathways, might counteract the global cytosolic ATP depletion during starvation in HeLa cells. Although due to the lack of liver kinase B1 (LKB1) in HeLa cells leading to a less active AMPK pathway [78,79], other energy stress signals might be initiated upon glucose removal. Such signaling might also be linked to ER-to-Golgi transport. However, additional experiments are required to understand the link between energy stress and the activity of the secretory pathway in this particular cancer cell model.

In this study, we also investigated the impact of energy stress induced by either glucose deprivation or the addition of 2-DG on subcellular Ca^2+^ homeostasis. Unexpectedly, cell treatment with 2-DG for 30 to 60 min, which evoked cellular ATP depletion within several minutes, did not lower the ER Ca^2+^ content of HeLa cells. Given the dependency of SERCA on ATP for counteracting the ER Ca^2+^ leak [66], it seems that under strong energy stress ER Ca^2+^ efflux is suppressed. While we did not further investigate possible mechanisms responsible for this phenomenon, we hypothesize that inhibition of protein synthesis, changes in phosphorylation patterns of ER channels and transporters, as well as signaling proteins of the unfolded protein response (UPR) might synergistically contribute to the preservation of the ER Ca^2+^ content under energy stress. Glucose removal also similarly affected Ca^2+^ homeostasis but less pronounced, which is in line with our findings on the effects of the different energy stresses on the subcellular ATP homeostasis, as well as vesicle motility. While 2-DG minimally affected basal ER Ca^2+^ levels, ER Ca^2+^ release and refilling were disturbed and cytosolic Ca^2+^ was significantly increased. We, then, investigated if the elevating effect of 2-DG on cytosolic Ca^2+^ contributes to the strong inhibitory effect of the antimetabolite on ER-to-Golgi transport. This could well be possible since elevated cytosolic Ca^2+^ immediately inhibits the motility of organelles including mitochondria [80] and vesicular structures [57], and long-term Ca^2+^ signaling has been shown to inhibit ER-to-Golgi transport of VSVG [41]. However, here, we demonstrated that even though Ca^2+^ elevation with a physiological agonist was as effective as treatment with 2-DG for hampering the overall motility of vesicular structures in the cell, ER-to-Golgi transport was only slightly affected by short-term Ca^2+^ mobilization as compared with 2-DG treatment. Therefore, it would appear that the inhibitory effects of energy stress on secretion were unlikely to be mediated by the disruption of Ca^2+^ homeostasis. Sargeant et al. [41] recently reported that long-term cytosolic Ca^2+^ signaling inhibited ER-to-Golgi transport in NRK cells, whereas, here, we reported a stimulation in transport caused by ATP in combination with BHQ (Figure 6f). Considering the different cell models (HeLa cells in this study versus NRK cells in [41]) and experimental conditions (i.e., short-term stimulation in this study versus long term in [41]) these studies are not in contradiction with one another. It is possible that different spatiotemporal patterns of cytosolic Ca^2+^ could cause different effects by acting on different or the same trafficking and regulatory machinery. However, additional research is essential to better understand the potential link between energy stress, Ca^2+^ alterations, and ER-Golgi transport in cancer cells.

## 5. Conclusions

In summary, our findings imply that the budding of transport vesicles at ER exit sites, their fusion to ER-to-Golgi intermediates, and further transfer of the fluorescent cargo constructs to the Golgi apparatus require ATP from glycolysis in the HeLa cancer cell model. Strikingly, initially, ER-to-Golgi transport is even facilitated in the absence of glucose and remains only minimally affected by Ca^2+^ mobilization for at least 15 min. Thus, we highlight that this initial and central step of the secretory pathway displays clear resilience to energy and Ca^2+^ stresses which improves our understanding of cancer biology and might have several implications in future cancer therapy.

## Supplementary Materials

The following are available online at https://www.mdpi.com/2073-4409/9/10/2311/s1, Figure S1: Schematic representation and example images for ER-to-Golgi transport measurements, Figure S2: Comparison of ER-to-Golgi transport efficiency of the soluble cargo construct (GFP-F_M_4-GH) and transmembrane cargo construct (GFP-F_M_4-VSVG_tm_) in HeLa versus NRK cells, Figure S3: ER-to-Golgi transport in NRK cells, Figure S4: Representative time course of a glucose-deprived HeLa cell showing ER-to-Golgi transport over time, Figure S5: ER-morphology is not changed upon short-term energy stress in HeLa cells, Figure S6: Severe energy depletion via 2-DG does not affect the microtubule network in HeLa cells, Figure S7: Induction of severe energy stress does not impair disaggregation of CAD-containing cargo constructs, Figure S8: ER-to-Golgi transport of transmembrane cargo in HeLa cells over 30 min, Video S1: Vesicle movement in HeLa cells treated with glucose-containing buffer, Video S2: Vesicle movement in HeLa cells treated with 2-DG-containing buffer, Video S3: Vesicle movement in HeLa cells after Ca^2+^-mobilization with buffer containing no Ca^2+^ and 0.1 mM EGTA, Video S4: Vesicle movement in HeLa cells after Ca^2+^-mobilization with buffer containing 2 mM Ca^2+^.
