# Supplementary figures and images for: ER-to-Golgi Transport in HeLa Cells Displays High Resilience to Ca2+ and Energy Stresses
Source: Cells. 2020 Oct 17;9(10):2311. doi: 10.3390/cells9102311 (PMC7603030; doi:10.3390/cells9102311)

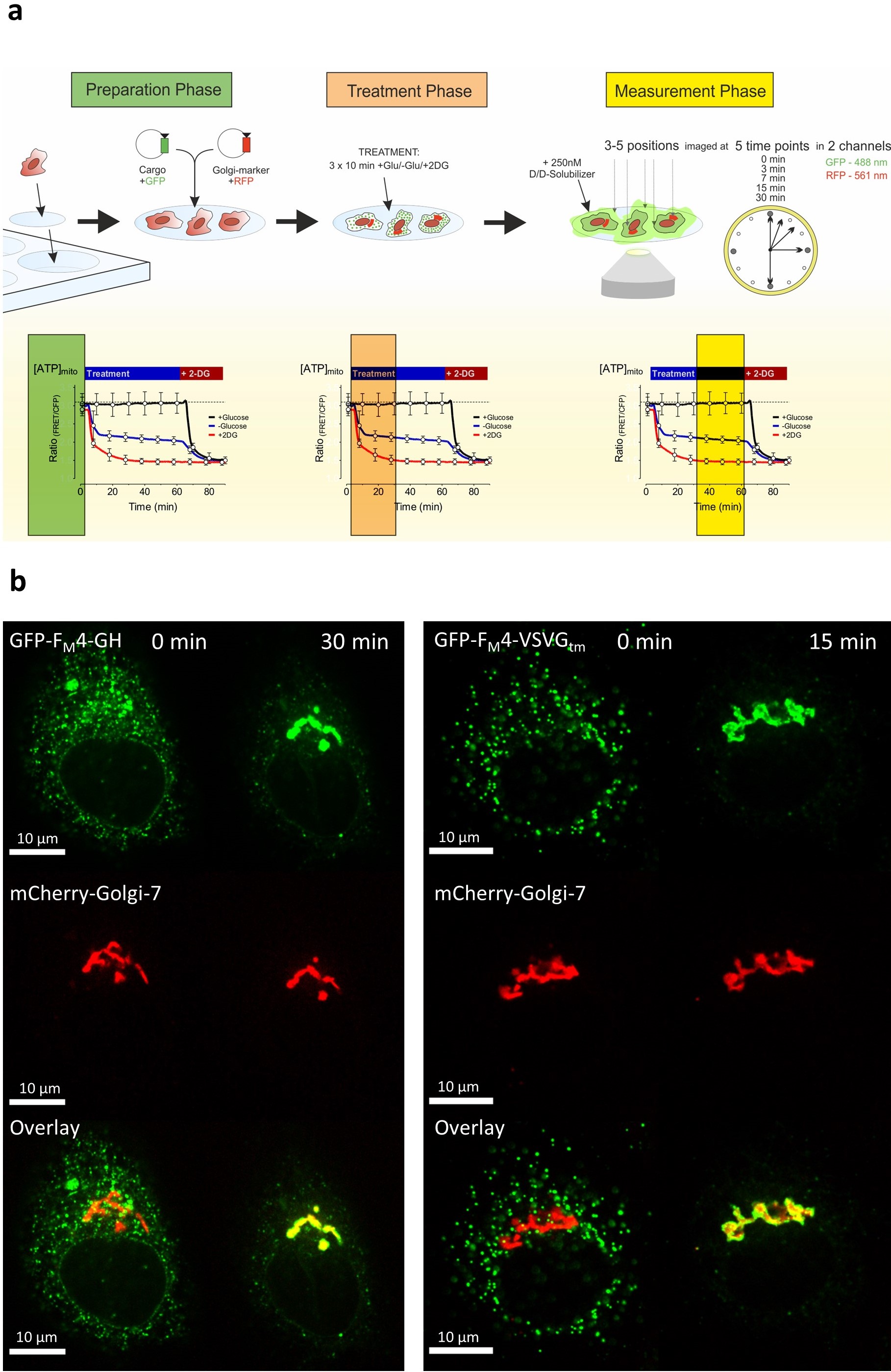

Supplement: Supplementary file 1 [file cells-09-02311-s001.zip › Supplementary Figures Revised/SupplementaryFigure1.JPG]

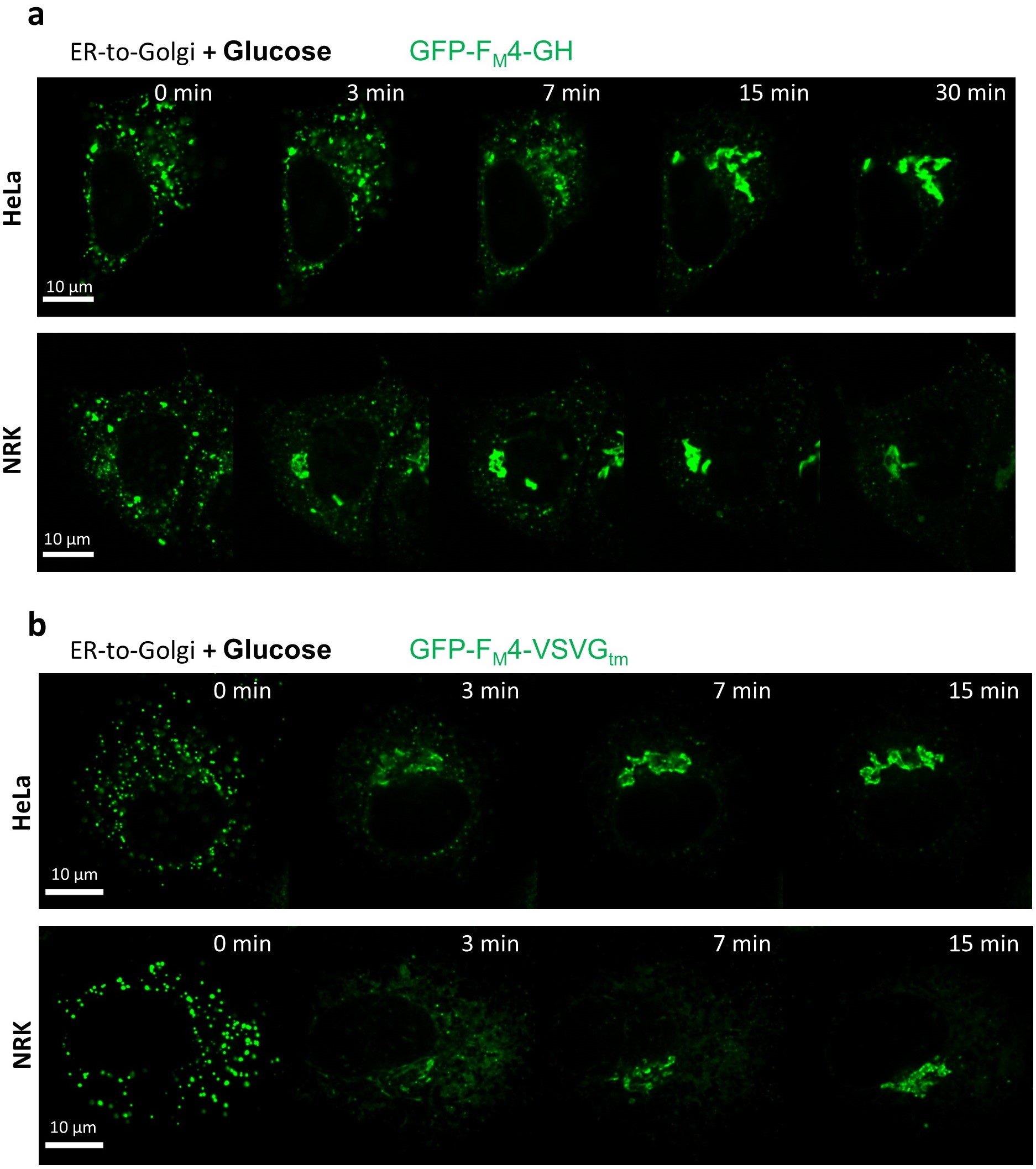

Supplement: Supplementary file 1 [file cells-09-02311-s001.zip › Supplementary Figures Revised/SupplementaryFigure2.jpg]

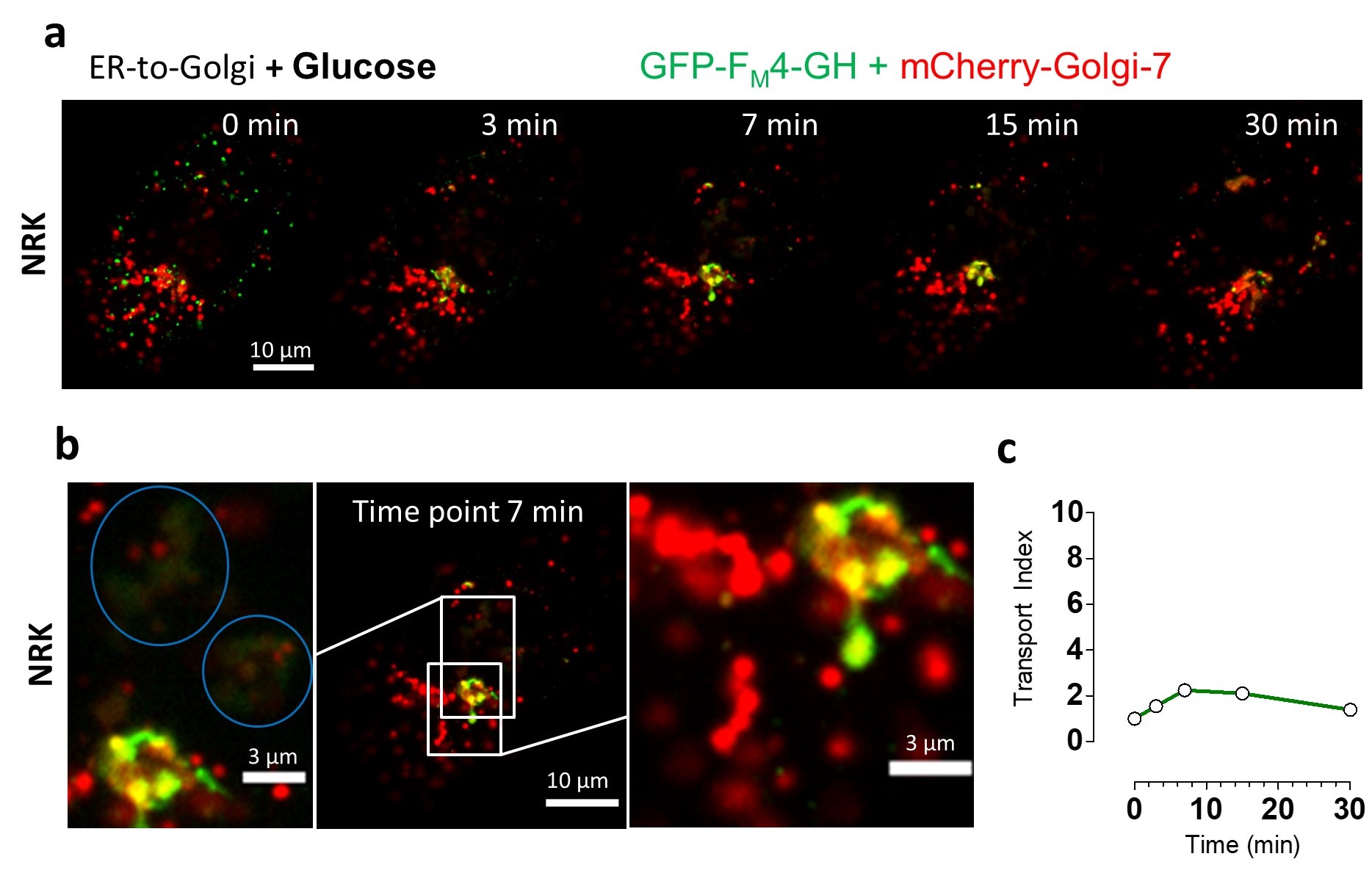

Supplement: Supplementary file 1 [file cells-09-02311-s001.zip › Supplementary Figures Revised/SupplementaryFigure3.jpg]

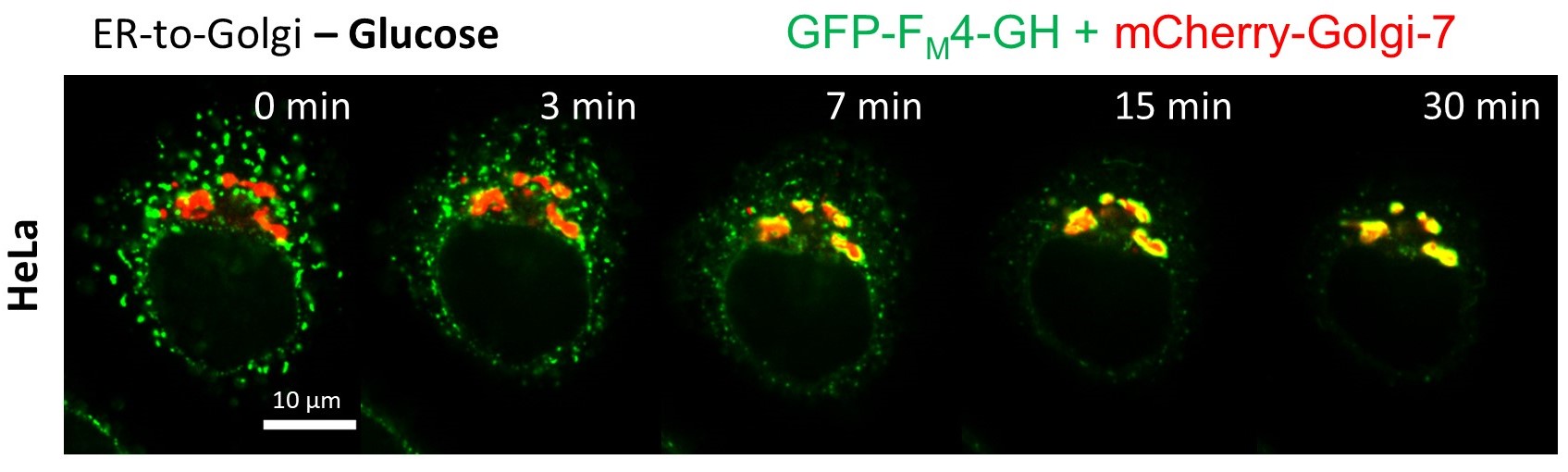

Supplement: Supplementary file 1 [file cells-09-02311-s001.zip › Supplementary Figures Revised/SupplementaryFigure4.JPG]

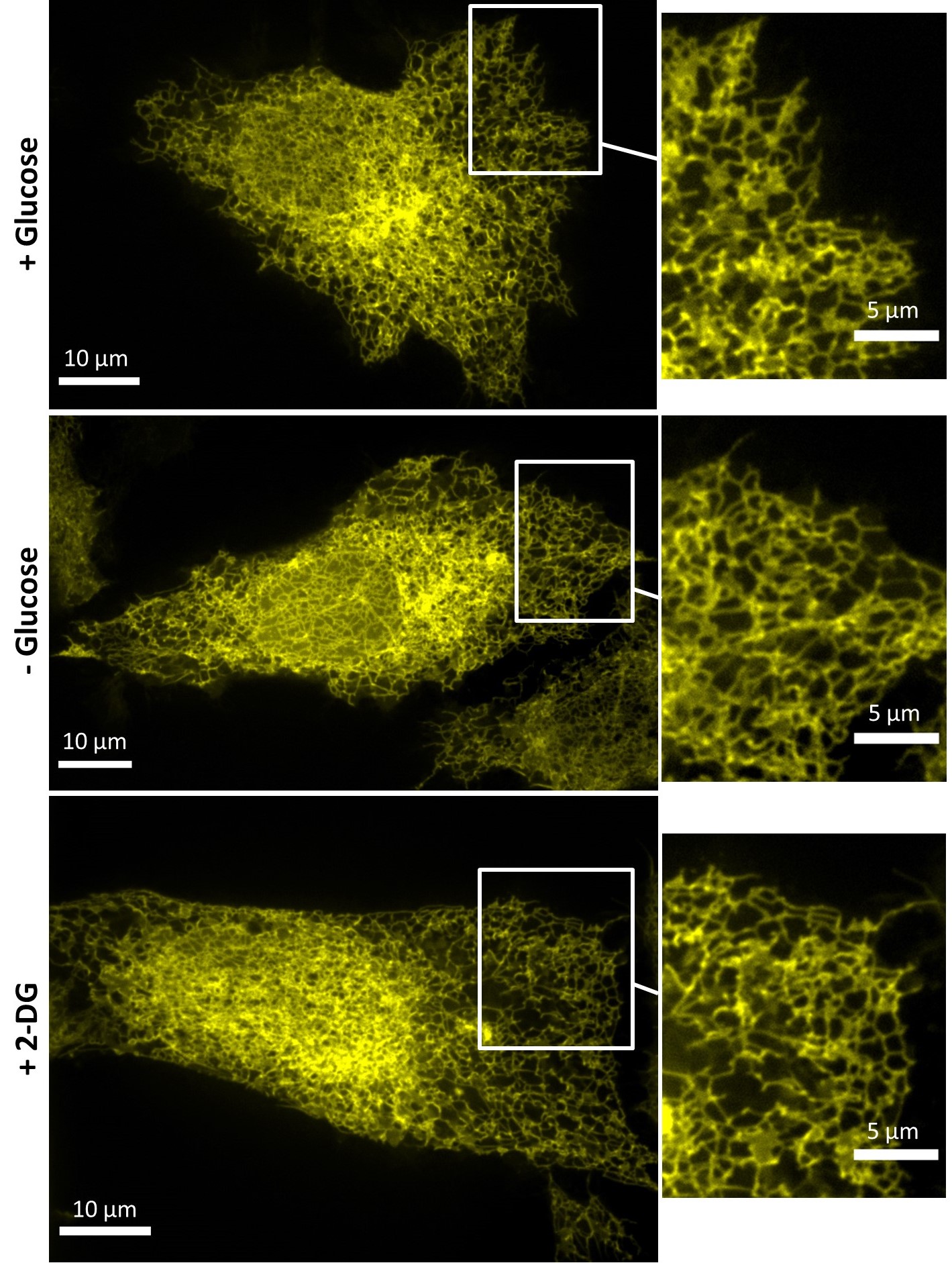

Supplement: Supplementary file 1 [file cells-09-02311-s001.zip › Supplementary Figures Revised/SupplementaryFigure5.JPG]

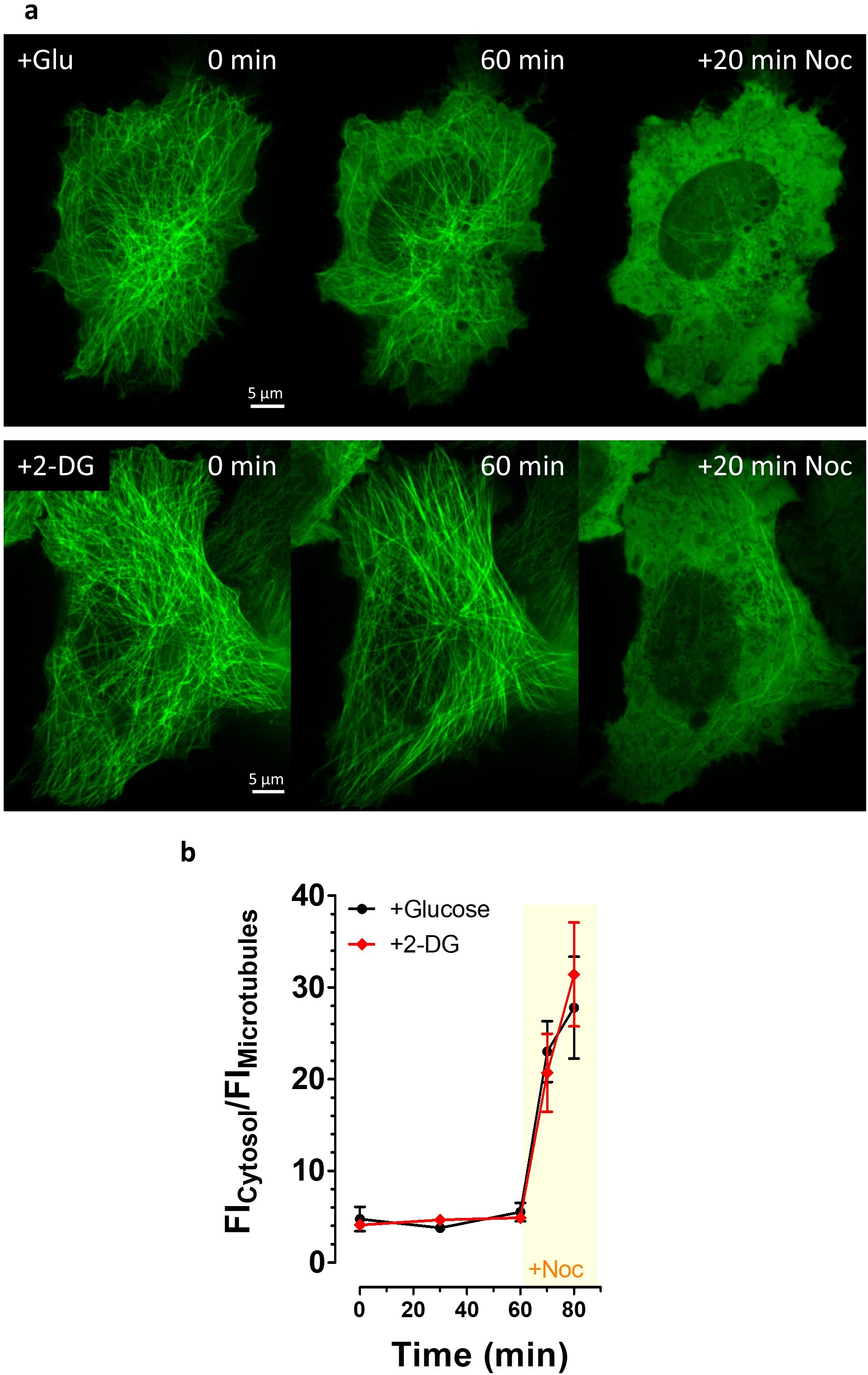

Supplement: Supplementary file 1 [file cells-09-02311-s001.zip › Supplementary Figures Revised/SupplementaryFigure6.JPG]

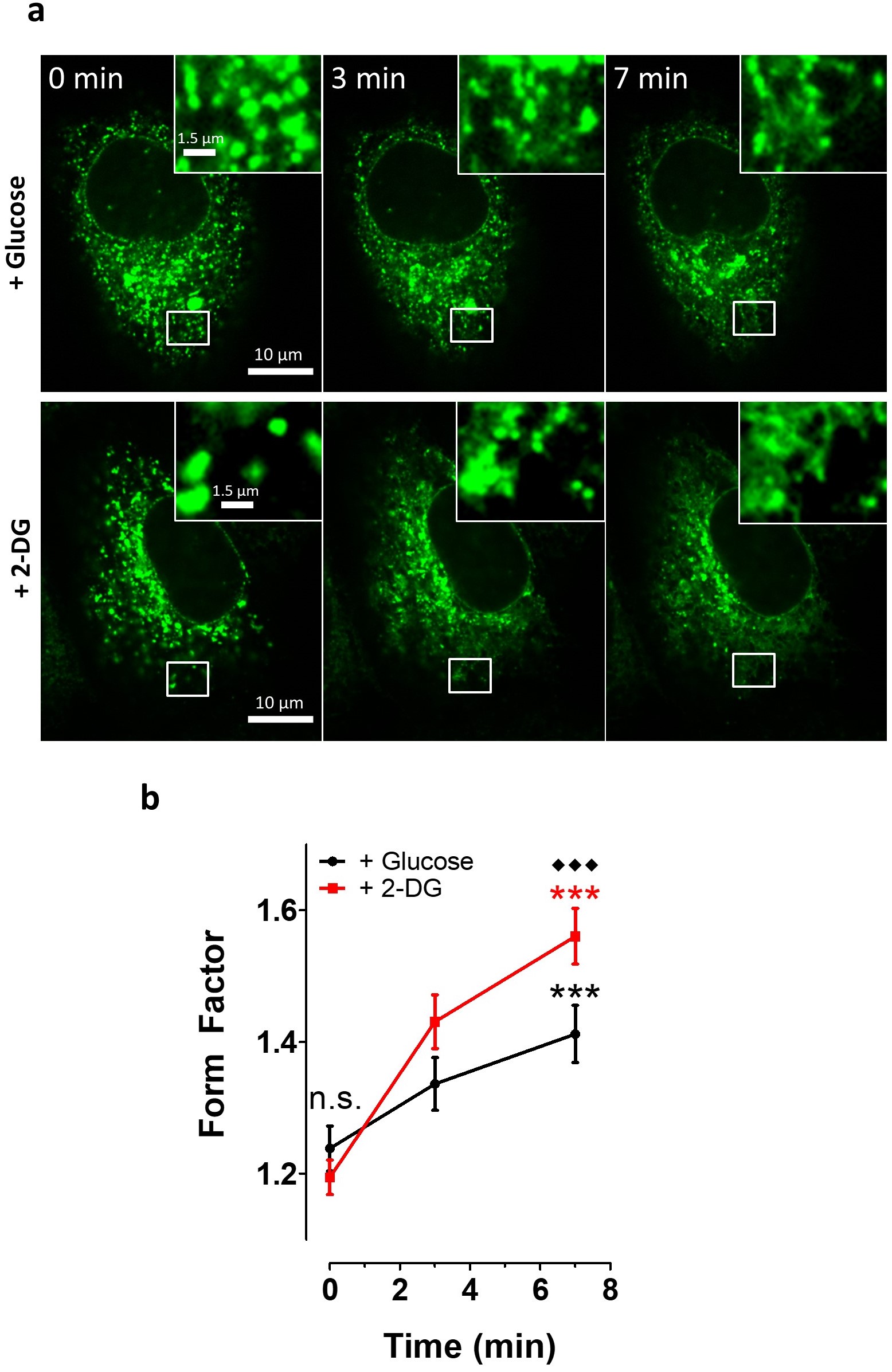

Supplement: Supplementary file 1 [file cells-09-02311-s001.zip › Supplementary Figures Revised/SupplementaryFigure7.JPG]

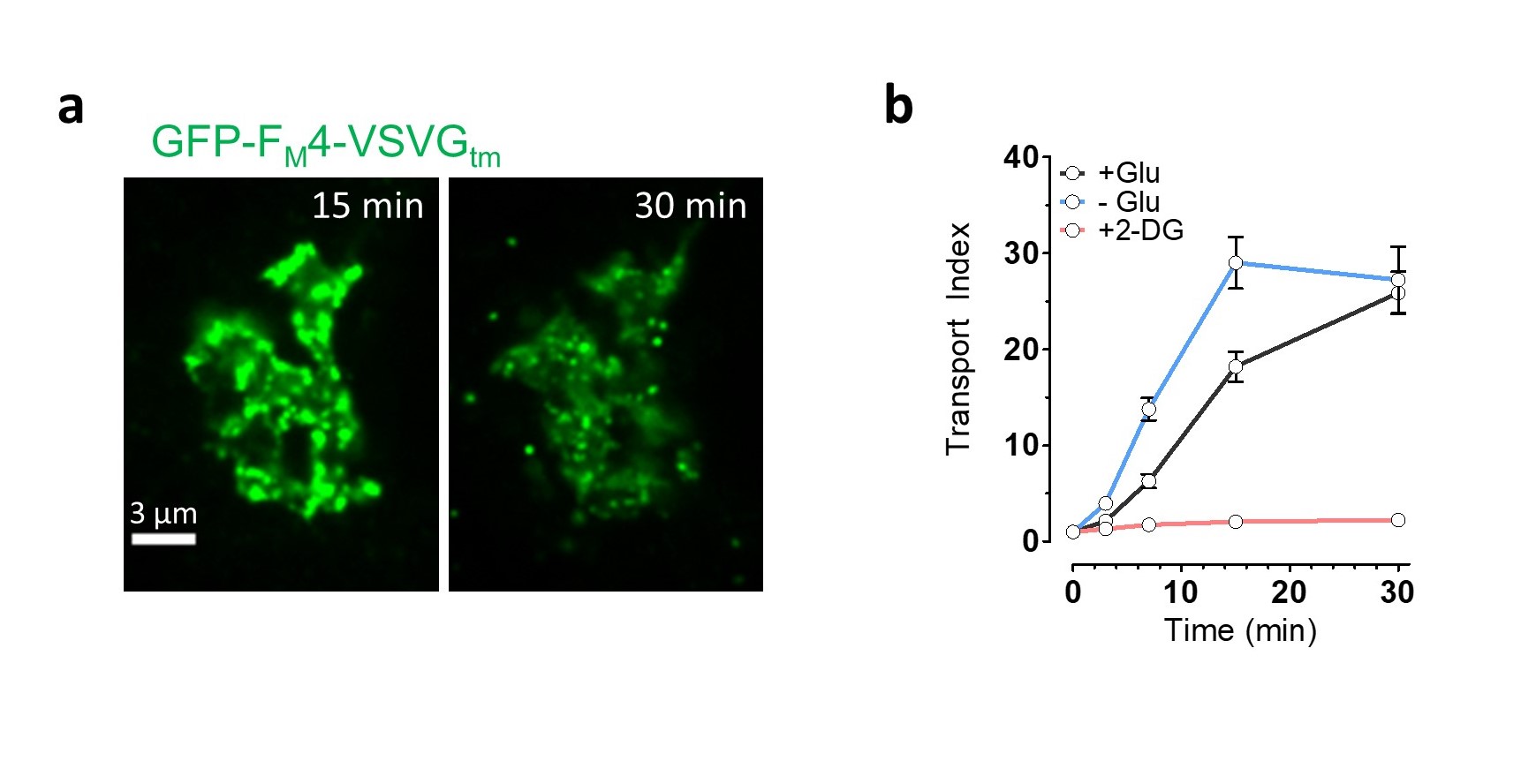

Supplement: Supplementary file 1 [file cells-09-02311-s001.zip › Supplementary Figures Revised/SupplementaryFigure8.JPG]
